# In Situ Ligand Transformation for the Development of Luminescent 3D Metal–Organic Frameworks with Diamond-like Topology

**DOI:** 10.1021/acs.cgd.4c01496

**Published:** 2025-01-22

**Authors:** Antonio A. García-Valdivia, Sara Rojas, Duane Choquesillo-Lazarte, Antonio Rodríguez-Diéguez, José Ángel García, Javier Cepeda, Pablo Salcedo-Abraira

**Affiliations:** † Departamento de Química Inorgánica, Facultad de Ciencias, Universidad de Granada, 18071 Granada, Spain; ‡ Laboratory for Crystallographic Studies IACT, CSIC-UGR, Av. Las Palmeras n°4, 18100 Granada, Spain; § Departamento de Física, Facultad de Ciencia y Tecnología, Universidad del País Vasco/Euskal Herriko Unibertsitatea (UPV/EHU), 48940 Leioa, Spain; ∥ Departamento de Química Aplicada, Facultad de Química, Universidad del País Vasco (UPV/EHU), Paseo Manuel Lardizábal 3, 20018 Donostia-San Sebastián, Spain

## Abstract

Here, the synthesis by a soft solvothermal route of two novel isoreticular compounds based on the in situ generated (by a nucleophilic aromatic substitution) 2-hydroxi-5-(trifluoromethyl)­pyrimidine (H_1_L) ligand and Zn­(II) and Cd­(II) as metallic centers (with the general formula [ML_2_]_n_ and labeled as GR-MOF-30 for M = Zn and GR-MOF-31 for M = Cd) is reported, together with their detailed structural and photoluminescent characterization. These metal–organic frameworks are the first examples of coordination compounds based on Zn­(II) and Cd­(II) constructed with this novel ligand. Structures show remarkable intermolecular interactions, including C–F···π and π···π stacking, which not only stabilize the structure but also improve the luminescent properties of the materials. DFT calculations were employed to unequivocally assign the bands observed in UV–vis solid-state spectroscopy. A photophysical study of the materials revealed that GR-MOF-30 and GR-MOF-31present fluorescence band maxima at 394 and 388 nm, respectively, with phosphorescence band maxima and emission lifetimes of 500 and 48.1 ms for **GR-MOF-30** and 450 and 69.2 ms for GR-MOF-31. Interestingly, the photoluminescence properties of compound GR-MOF-30 are hardly affected by the change of temperature; meanwhile, GR-MOF-31 shows a transition from a dominating fluorescent emission at room temperature to a phosphorescent emission at 25 K.

## Graphical abstract

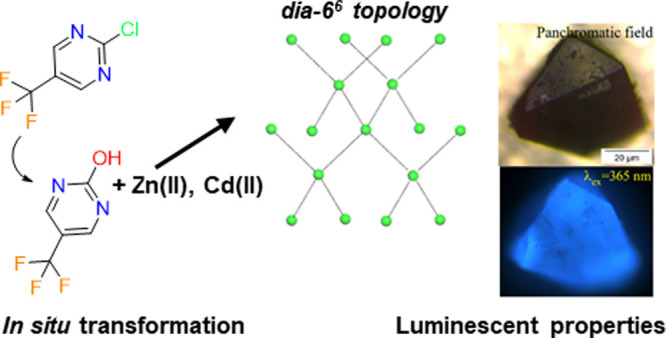

## Introduction

Coordination chemistry has greatly advanced the development of new metal–organic materials, namely, porous coordination polymers or metal–organic frameworks (MOFs), to deal with the problems that currently concern society, such as the search for better agrochemicals,^1,2^ the elimination of contaminants,^3,4^ the development of new drugs,^5,6^ or sensing a wide range of hazardous molecules.^7–9^ Through an appropriate selection of the building blocks (metal ions and organic ligands), the crystal structure of MOFs can be predesigned almost at will, achieving the desired structural characteristics that may in turn lead to specific properties.^10–12^ It is well-known that nitrogen-containing heterocycles are commonly used as ligands due to their good coordination capacity, especially with M­(II) ions such as Zn­(II) or Cd­(II). With regard to the design of MOFs, pyrimidine-like ligands have largely shown promising capacity to render not only high-dimensional structures but also, most importantly, strongly bonded frameworks benefiting from high thermal and chemical stability.^13^ Moreover, owing to the relatively angular planar disposition of the nitrogen-donor atoms, pyrimidine-based derivatives are appropriate ligands to yield, when combined with metal ions, fascinating structures with topologies resembling those of the inorganic zeolites, in contrast with other N-donor ligands.^14,15^ In particular, MOFs possessing 4-connected nodes linked by linear linkers may also lead to diamond-like networks, characterized by their framework hardness and stability.^16^

Apart from the abovementioned structural characteristics, the use of aromatic N-donor ligands can also be appropriate in the design of MOFs with excellent luminescent properties.^17–19^ Recent research has shifted to studies focused on coordination polymers based on group d^10^ metal ions due to their capability to become stable long-lasting phosphorescence (LLP).^11,20–23^ This behavior is derived from the inherent properties of the organic ligand, which, in combination with these kinds of metal ions (ions possessing closed-shell electronic configuration), brings additional stability to the excitons of the ligand molecules by diminishing the habitual quenching derived from thermal vibrations, while they also allow for the occurrence of additional transitions based on ligand-centered (LCCT) or ligand-to-metal charge transfers (LMCTs).^24–26^ In the quest for novel photoluminescent MOFs with enhanced emission capacity, i.e., more efficient luminescence, the partial replacement of hydrogen by fluorine atoms has recently been shown to bring important benefits to the frameworks in terms of photoluminescence efficiency, due to the rigidification of the chromophores, for instance, by reducing the spinning ability of usual rotamers, such as methyl groups.^27^

Herein, we report the synthesis and structure of two novel MOF materials based on the pyrimidine-containing −CF_3_ group 2-hydroxy-5-(trifluoromethyl)­pyrimidine (H_1_L) as a linker and group 12 metal ions (M = Zn­(II) and Cd­(II)). H_1_L was in situ derived from 2-chloro-5-(trifluoromethyl)­pyrimidine. In situ ligand reactions during the synthesis of MOFs or coordination compounds under solvothermal conditions are widely described in the literature.^28–31^ Thanks to its delocalized electron density, this ligand is a great candidate for enhancing emissive properties. These materials corroborate two development paths: (1) the potential of pyrimidine derivative linkers to construct new coordination polymers with interesting properties and (2) the use of in situ reactions to search for new ligands with enhanced properties. Our results confirm that these materials present interesting luminescence properties, especially the Cd-based material that exhibits long-lasting phosphorescence.

## Materials and Methods

### Reagents

All of the chemicals were of reagent grade and used as commercially obtained.

### Synthesis

#### [ML_2_]_*n*_ (M = Zn­(II) for GR-MOF-30 and Cd­(II) for **GR-MOF-31**

Colorless single crystals were obtained following the next synthesis method for both materials. 0.055 mmol (10.00 mg) of 2-chloro-5-(trifluoromethyl)­pyrimidine was dissolved in 0.5 mL of DMF; then, 0.5 mL of distilled water was added. On the other hand, 0.055 mmol of the corresponding metal acetate (M­(C_2_H_3_O_2_)_2_, 10.09 mg for **GR-MOF-30** and 12.65 mg for **GR-MOF-31**, was weighed in a vial and dissolved in 1 mL of a solvent mixture (0.5 mL of distilled water and 0.5 mL of DMF). The ligand and metal solutions were mixed, and the resulting solution was placed in a closed glass vessel and introduced into an oven at 95 °C for 24 h, with the obtained crystals recovered by filtration (yield = 15%). The synthesis was successfully scaled up by five times (from 10 mg of ligand to 50 mg). During the synthesis, an in situ nucleophilic aromatic substitution (S_N_Ar) process occurs to yield the H_1_L ligand (Figure 1).

**Figure 1:**
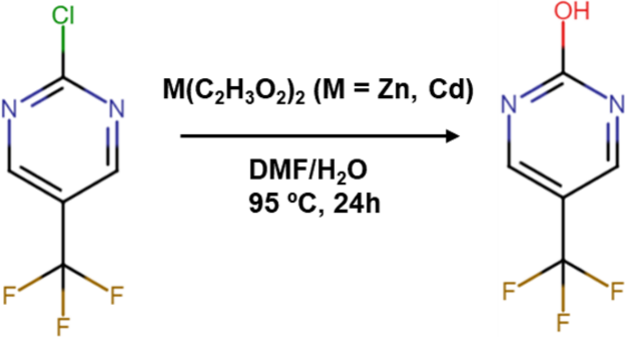
In situ generation of the H_1_L ligand through a nucleophilic aromatic substitution (S_N_Ar) reaction.

### Crystallographic Studies

X-ray data collection of suitable single crystals of all compounds was done at 100(2) K on a Bruker VENTURE area detector equipped with graphite-monochromated Mo Kα radiation (λ = 0.71073 Å) by applying the ω-scan method. The data reduction was performed with the APEX2 ^32^ software and corrected for absorption using SADABS.^33^ Crystal structures were solved by direct methods using the SIR97 program^34^ and refined by full-matrix least-squares on *F* ^2^ including all reflections using anisotropic displacement parameters, by means of the OLEX software using the SHELX crystallographic package.^35–37^ Details of the structure determination and refinement of **GR-MOF-30** and **GR-MOF-31** are summarized in Table S1. It should be noted that for **GR-MOF-30**, the disorder of the −CF_3_ groups is generated by symmetry operations. Crystallographic data (excluding structure factors) for the structure reported in this paper have been deposited with the Cambridge Crystallographic Data Centre as supplementary publication nos. CCDC 2388248 and 2388249 (for **GR-MOF-30** and **GR-MOF-31**, respectively). Copies of the data can be obtained free of charge on application to the Director, CCDC, 12 Union Road, Cambridge, CB2 1EZ, U.K. (Fax: + 44–1223–335033; e-mail: deposit@ccdc.cam.ac.uk or http://www.ccdc.cam.ac.uk).

### Physicochemical Characterization

Powder X-ray diffraction (PXRD) patterns were collected for polycrystalline samples in a Philips X’PERT powder diffractometer equipped with Cu–Kα radiation (λ = 1.5418 Å) over the 5 < 2θ < 50° range with a step size of 0.026° and an acquisition time of 2.5 s per step at 25 °C. PXRD patterns were also collected in a BRUKER D8 DISCOVER equipped with Cu–Kα radiation (λ = 1.54059 Å) in a 2θ range of 4–50° with a 2° step size and 50 s of acquisition time. Le Bail profile fitting analysis was made using the FULLPROF program^38^ based on the space group and cell parameters obtained from single-crystal X-ray diffraction. Thermogravimetric analyses were performed on a SHIMADZU TGA-50H equipment under air with a heating rate of 10 °C·min^–1^. For the humidity-dependent stability test, the samples were kept at 30 °C and 70% relative humidity (RH) in a Memmert HHP 108 climatic chamber for 24 h.

### Photoluminescence Measurements

Photoluminescence excitation and emission spectra were acquired on an Edinburgh Instruments FLS920 spectrometer. All measurements were conducted under high vacuum (about 10^–8^ mbar) to avoid the presence of oxygen or water in the sample holder. An IK3552R-G HeCd continuous laser (325 nm) and a Müller-Elektronik-Optik SVX1450 Xe lamp were employed as excitation sources for the steady-state measurements, whereas a pulsed light-emitting diode (LED, λ = 340 nm) or laser diode LDH-P-C-375 (λ = 375 nm) was used for the decay curves. The fluorescence counts were detected on a photomultiplier tube (PMT) coupled to the spectrometer. Absolute quantum yields were measured on solid samples by means of a Horiba Quanta-φ integrating sphere coupled to an Oriel Instruments MS257 lamp as the excitation source and a Horiba iHR550 spectrometer to analyze the emission, both of them by optical fiber. A calibration pattern was applied to correct the emission spectra before signal integration, and five measurements were accomplished to properly estimate the mean and standard deviation. Photographs of irradiated samples were acquired at room temperature in a micro-PL system equipped in an Olympus optical microscope under Hg lamp illumination. Videos of the emission afterglow were recorded directly at the sample holder of the cryostat. Photostability measurements were recorded at the Edinburgh Instruments FL980 spectrometer with its abovementioned setup using continuous irradiation of the IK3552R-G He–Cd continuous laser at λ_ex_ = 325 nm, setting an accumulation time step of 2 s for each individual acquisition.

### Computational Details

In order to simulate the properties of the MOFs, a finite fragment consisting of a pseudotetrahedral complex (based on a central zinc atom coordinated to four complete ligands) cut from the X-ray diffraction structure was employed. In this fragment, we replaced the surrounding zinc atoms (neighboring metal centers) with lithium ions so that the fragment could preserve its original arrangement from the extended structure in the MOF. All calculations were performed at density functional theory (DFT) with the Gaussian 16 program package (revision A.03)^39^ using the 6-31G** basis set for C, N, O, H, F, and Li atoms and LANL2DZ for the Zn atom.^40,41^ The geometry was optimized with the hybrid exchange-correlation functional B3LYP^42^ in several steps, wherein the adequate positions of terminal Li atoms were found by freezing the whole [ZnL_2_]_4_ complex and only optimizing those atoms, and fixing the Li atoms to leave all atoms of the zinc complex free in a second step. All optimizations were accompanied by additional calculations of the vibrational frequencies in both the ground and excited state geometries, thanks to which the nature of the minima and the absence of imaginary frequencies at the converged geometries were confirmed. Time-dependent DFT (TD-DFT) calculations at the same level of theory but using the hybrid exchange-correlation functional CAM-B3LYP^43^ were performed from the optimized molecular geometry of the ground state, and 100 vertical electronic transitions were computed. The fluorescence emission energy from each optimized excited state was calculated by subtracting the energy of the excited state from the energy of the ground state at the optimized geometry of the former. The D3 version of Grimme’s dispersion correction (GD3 zero damping factor) was included in these calculations.^44^

## Results and Discussion

### Synthesis of [ML_2_]_*n*_ (M = Zn­(II), Cd­(II))

Both compounds were synthesized by using a simple solvothermal method (see Materials and Methods for further details). Briefly, two H_2_O:DMF solutions containing the ligand and the corresponding metallic acetate were mixed and heated at 95 °C for 24 h, giving colorless crystals suitable for single-crystal X-ray diffraction. It should be noted here that diverse attempts to increase the yield of the reaction (15%) were made. Longer reaction times (up to 96 h) did not lead to higher yields. Also, the addition of an additional acid (HCl) or base (NaOH, Et_3_N) to the media resulted in the failure of the reaction. Considering the general mechanism of the S_N_Ar (see ESI, Scheme S1), one could hypothesize that the acetates in the media (from the metallic salts) are responsible for the hydroxyl generation required for the reaction. The addition of acid will then hamper the reaction; meanwhile, the addition of an extra-base would precipitate the M­(OH)_2_ species (as observed experimentally). To confirm this hypothesis, the reaction was also performed using different metallic precursors such as MCl_2_, M­(NO_3_)_2_, and MSO_4_, resulting in all the cases in the failure of the reaction, thus confirming our hypothesis

### Description of GR-MOF-30 [ZnL_2_]_*n*_

**GR-MOF-30** consists of a three-dimensional compact framework crystallized in the *I*4̅2*d* tetragonal space group (Table S1), wherein Zn­(II) ions are coordinated to four ligand molecules by one of their pyrimidine nitrogen atoms (N1) (Figure 2). The metal coordination sphere is described as a less distorted tetrahedron with N1 atoms located at the vertices, as corroborated by the continuous shape measurements (CShMs, S_T_ = 0.081). The bond distances of the coordination sphere and angles are gathered in Table S2. As detailed there, all the N–Zn–N bond angles are very similar and close to 108° in good agreement with an almost regular polyhedron.

**Figure 2:**
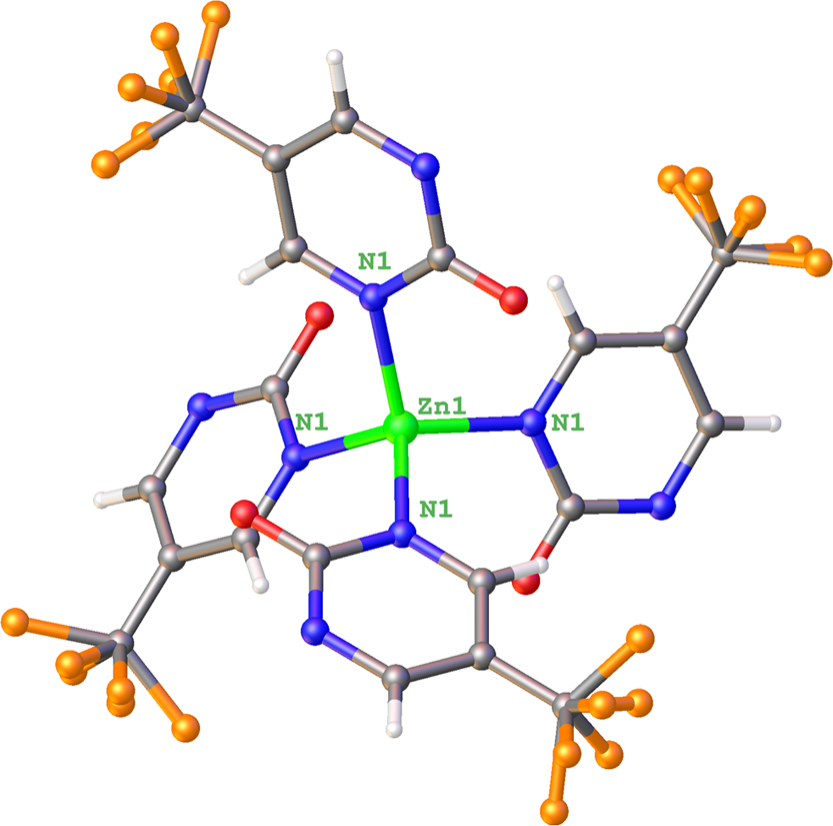
View of a fragment of the polymeric structure of GR-MOF-30 showing its tetrahedral coordination environment and four surrounding ligands. Zn, O, F, N, C, and H are represented in green, red, orange, blue, gray, and white, respectively. Note here that the −CF_3_ groups are disordered due to symmetry operations.

The MOF consists of a three-dimensional diamond-like structure where the metal centers are linked to other four neighboring centers by means of the bridging L ligands, which employ both of their pyrimidine nitrogen atoms to connect the metal ions to one another. In fact, the analysis of the structure with the TOPOS software confirms the (6^6^) point symbol of the diamond (**dia** topology) network (Figure 3).^45^ Therefore, it consists of a compact framework with channels through the *a* and *b* axes. However, the distribution of the ligands along with the CF_3_ groups' orientation toward these channels hinders the access to the pores (Figure S1), as confirmed by Platon calculations (0% of free volume) and TGA, where no mass loss of guest solvent molecules was observed (Figure S2). At a supramolecular level, there are no remarkable intermolecular interactions to be mentioned in the framework, a fact that suggests it could potentially benefit from flexible character. The purity of the material was corroborated by the Le Bail profile fitting of the powder X-ray diffraction (PXRD) patterns (Figure S3 and Table S3).

**Figure 3:**
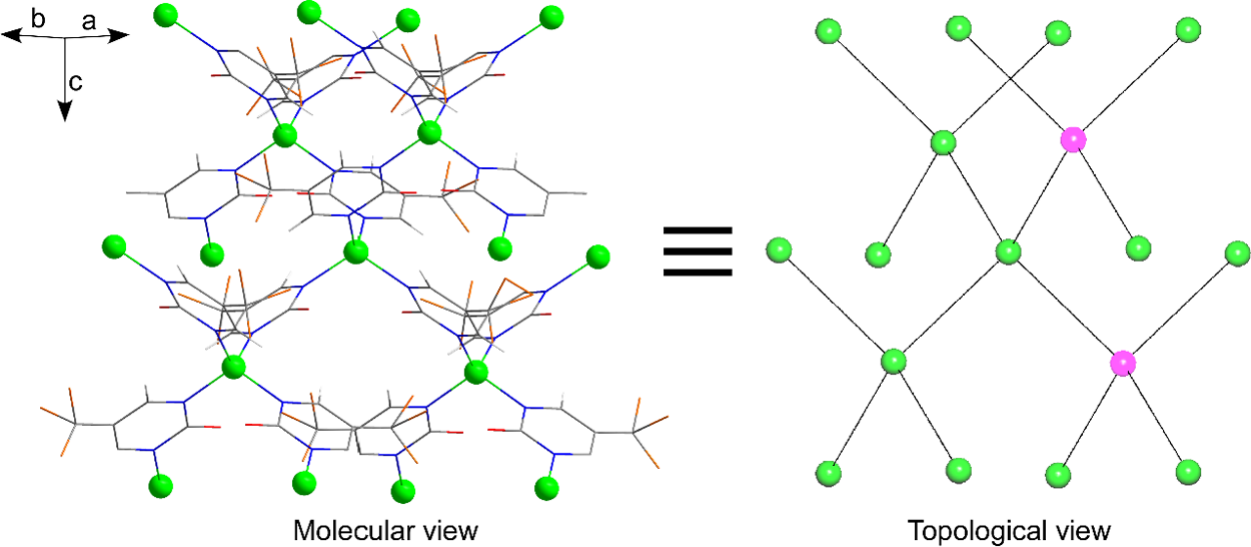
Comparative view of the molecular and topological framework of **GR-MOF-30**. Note that ligands act as linear linkers from a topological point of view.

### Description of GR-MOF-31 [CdL_2_]_*n*_

**GR-MOF-31** consists of a three-dimensional compact framework that crystallized in the *Pna*2_1_ orthorhombic space group (Table S1), wherein Cd­(II) ions exhibit the same tetrahedral-like coordination sphere established by four nitrogen atoms (N1, N2­(i), N3, and N4­(i)) pertaining to the two crystallographically independent ligands and their copies (Figure 4). In the present case, it is worth noticing that the coordination sphere is more distorted with regard to an ideal tetrahedron (S_T_ = 0.891 according to CShMs, see Table S4), primarily due to the larger size of the metal ion, which allows higher plasticity in the N–Cd–N angles (ranging from 96.66(8) to 120.68(8)°), although all coordination bond distances are quite regular (Cd–N being in the 2.237(2) to 2.299(2) range, see Table S5). A visual comparison of the coordination fragments of both compounds can be inferred from the overlay of their tetrahedral building units, i.e., [ML_4_]^2–^ coordination excerpts (see Figure S4).

**Figure 4:**
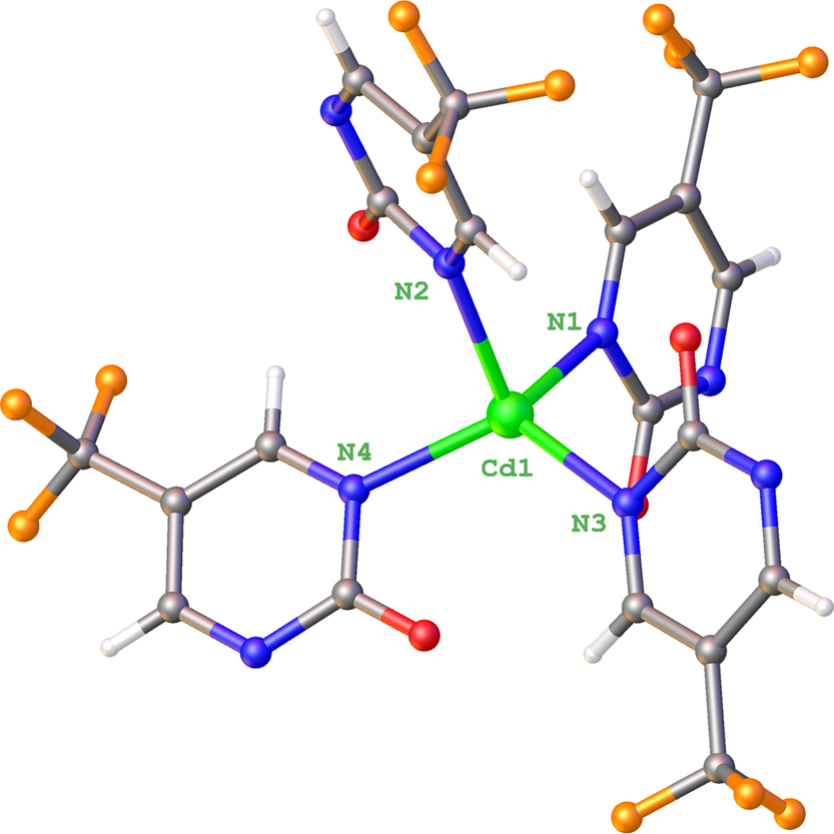
View of the secondary coordination sphere of **GR-MOF-31** showing the ligands and the numbering scheme for the atoms of the coordination center. Cd, O, F, N, C, and H are represented in green, red, orange, blue, gray, and white, respectively.

This compound presents an isoreticular network of that described for **GR-MOF-30**, taking into account that both the metal center shape and ligand connectivity are maintained. Nonetheless, the mentioned distortions at the coordination polyhedron generate slight changes in the framework with respect to the framework of **1** (Figures S5 and S6, where the same projections of the structures can be observed from different directions), which defines the relative orientation of the ligands and the interactions taking place among them. In particular, the present structure is characterized by the occurrence of some weak contacts between the CF_3_ groups and the aromatic ring of the L ligand, since the former are arranged perpendicularly and point to the center of the π cloud of the ligand at a distance of ca. 3.28 Å (Figure S7).^39,40^ Despite these differences, **GR-MOF-31** was dense as in the case of **GR-MOF-30** **1**, confirmed again by Platon (0% free volume) and TGA (Figure S8). Finally, the purity of the material was corroborated by the Le Bail profile fitting of the PXRD patterns (Figure S9 and Table S3).

### Thermal and Humidity-Dependent Stability

The thermal and structural stability under high relative humidity (RH = 70%) of both compounds was assessed by TGA and PXRD. TGA under air of **GR-MOF-30** (Figure S2) showed almost no weight loss (in agreement with Platon calculations and confirming the dense character of the solid) up to 400 °C, above which the decomposition of the organic ligand takes place. In the same way, the TGA of **GR-MOF-31** (Figure S8) showed no weight loss until 410 °C, when the ligand started to decompose. The chemical stability upon humidity was evaluated by exposing the samples at 70% RH and 30 °C for 24 h. PXRD (Figures S10 and S11) revealed no significant changes in the structures of both compounds, evidencing their chemical stability.

### Photophysical Study

In view of the potential photophysical properties of **GR-MOF-30** and **GR-MOF-31**, given their aromatic ligands and closed-shell metal ions,^46^ diffuse reflectance measurements were performed on the compounds to check their absorption capacity in the solid state. The spectra of both compounds show an almost indistinguishable pattern consisting of a main absorption band peaking at 332 nm, in addition to a less intense shoulder whose maximum is centered at 255 nm (see Figure S12). Although the nature of the compounds allows to unambiguously attribute the bands to electronic excitations occurring on the ligands, TD-DFT calculations were conducted on a suitable model of **GR-MOF-30** (model 1 hereafter) as a representative counterpart for both compounds (due to its lower electron density) in order to properly assign the transitions (see Computational Details for further information and Figure S13). The calculated spectrum reproduces quite well the shape of the experimental one, showing a blue shift of about 30 nm (as usually observed for these kinds of calculations, probably due to the simplicity of the finite model employed compared to the real polymeric structure), although it fails to reproduce the intensity of the bands (they show opposite relative intensities compared to the experiment, Figure 5). In any case, it can be assured that the absorption process is governed by two well-distinguished singlet-to-singlet processes: (i) the dominant band of the spectrum (experimentally peaking at 332 nm) is mainly derived from the occurrence of two ligand-centered excitations occurring at 316 and 298 nm, wherein the first represents the HOMO → LUMO transition, whereas the second one is majorly described by the HOMO–3 → LUMO + 1 transition, to reach the first and the eighth excited states, respectively; (ii) the second (highest-energy) shoulder located at 255 nm is represented by the theoretical band situated at 232 nm, which is mainly composed of the HOMO–2 → LUMO + 8 excitation that gives rise to the 65th excited state (Table S6).

**Figure 5:**
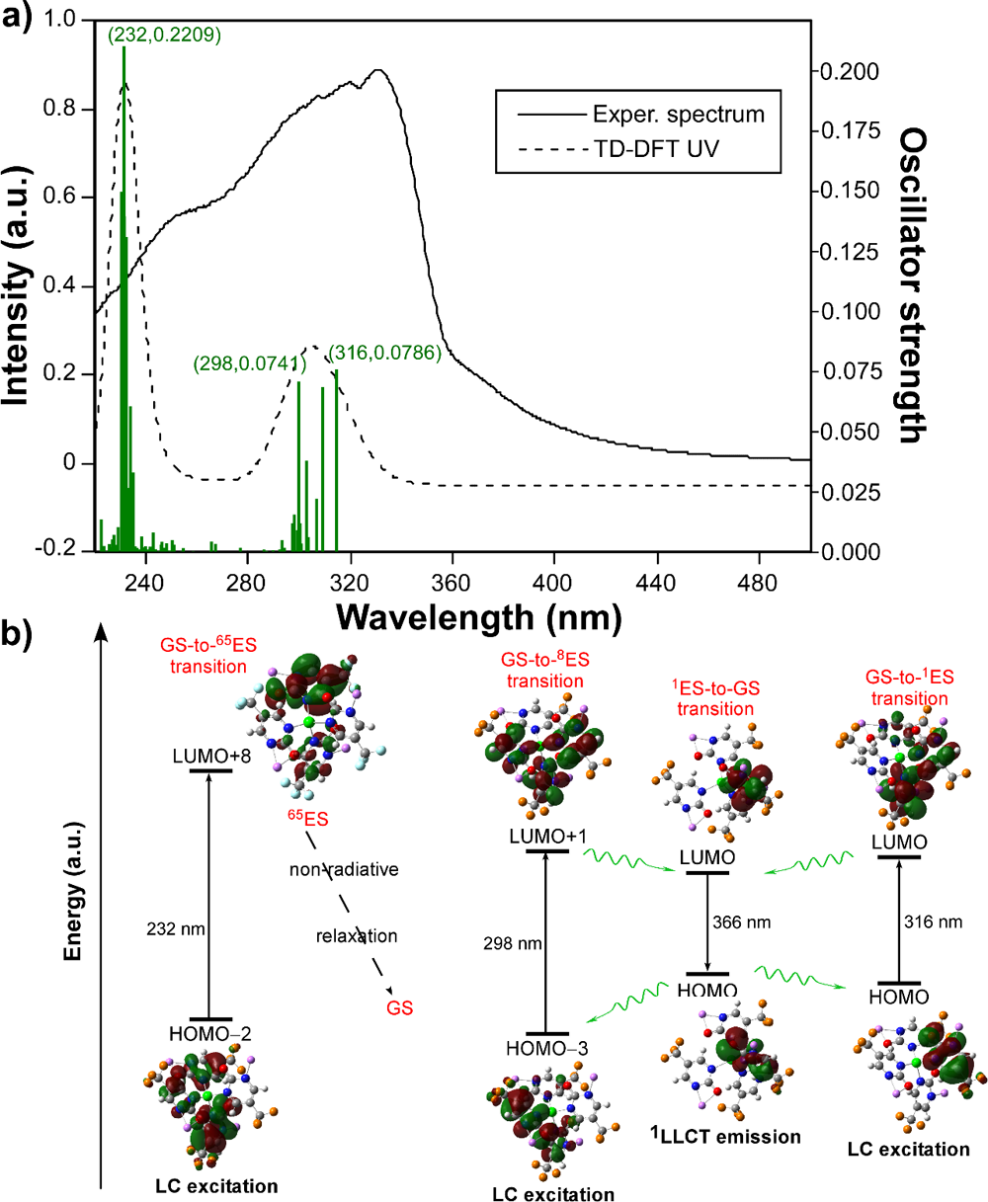
TD-DFT calculations on the model of **GR-MOF-30** represent the most important electronic transitions governing the photoluminescence process.

### Photoluminescence Properties

Photoluminescence properties of **GR-MOF-30** and **GR-MOF-31** were measured by using a polycrystalline sample at variable temperatures. At room temperature and under high vacuum (of ca. 3 × 10^–6^ bar), both compounds present a very similar luminescence pattern. Under 325 nm monochromatic excitation, **GR-MOF-30** shows a wide band centered at 394 nm with a lower intensity shoulder of similar wideness, with the maximum at 500 nm (Figure 6a). A similar spectrum is observed for **GR-MOF-31**, showing the band maximum at 390 nm and a shoulder at 450 nm (Figure 6b).

**Figure 6:**
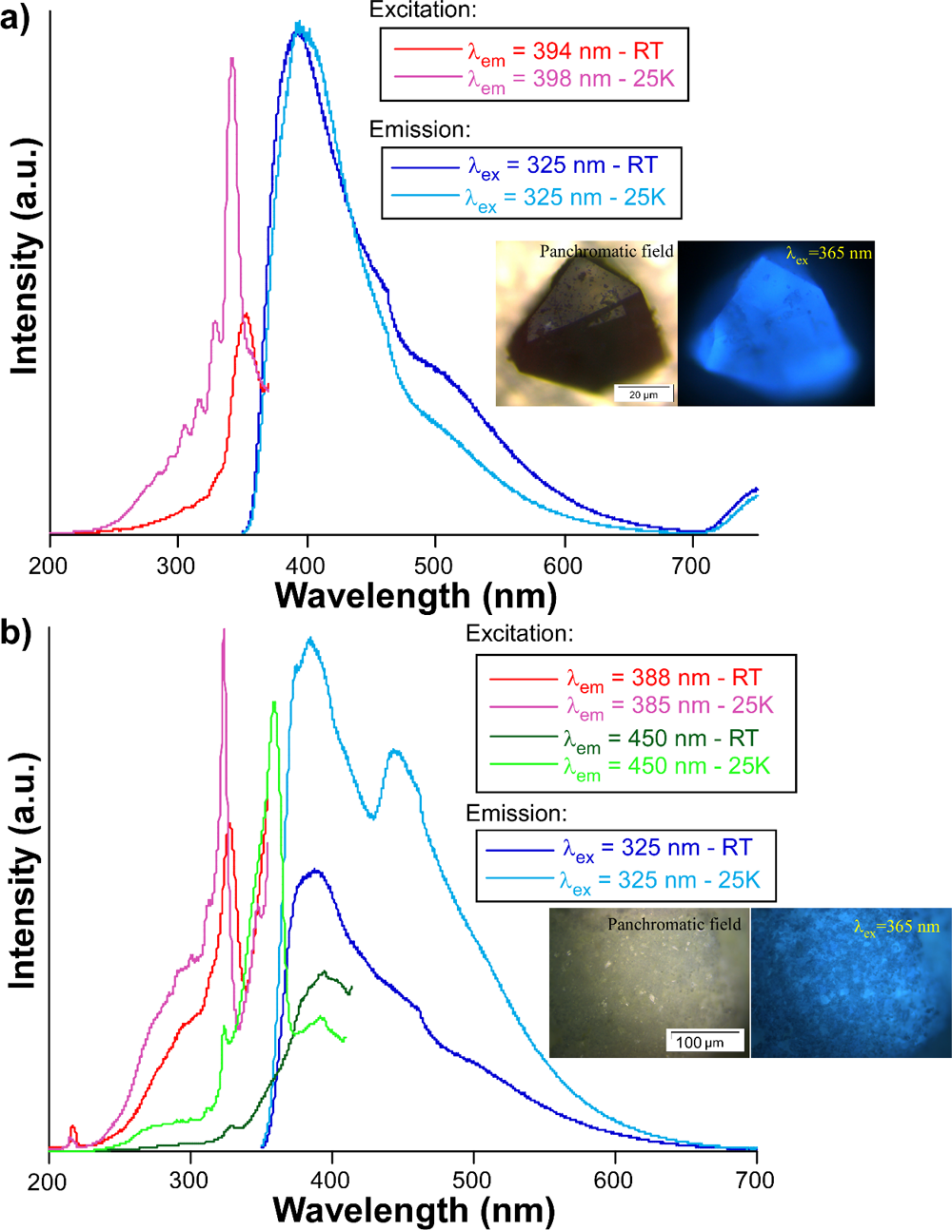
Variable temperature steady-state photoluminescence excitation and emission spectra recorded under vacuum for polycrystalline samples of: (a) **GR-MOF-30** and (b) **GR-MOF-31**. Insets show microphotoluminescence images taken on a microscope for both samples.

The excitation spectrum recorded at the most intense emission band (λ_em_ = 394 nm for **GR-MOF-30** and 390 nm for **GR-MOF-31**) shows the occurrence of a main band sited at 340 nm dominating the spectrum, in addition to some other lower intense contributions in the 285–335 nm range, thus meaning that the emission process is related with the most intense (experimental) absorption process coming from the first and eighth singlet excited states (S_1_ and S_8_ hereafter, respectively). Instead, the absence of any significant band in the 255 nm region suggests that the high-energy absorption band is not involved in the photoluminescence process of the compound because the 65th excited state (S_65_) either promotes a nonradiative relaxation or transfers the energy to the lowest-lying excited states. In order to gain further insights into the photoluminescence mechanism of the compounds, the geometries of the S_1_ and S_8_ states of model 1 have been optimized at the same TD-DFT level. Compared to the optimized fragment at the ground state, the optimized structure at the S_1_ state reveals that the coordination bond distances are shortened, and the coordination sphere is subtly reorganized by mainly regularizing the N–Zn–N angles, in such a way that a more regular tetrahedron is observed for the S_1_ state (S_T_ = 0.161 for S_1_ vs 0.304 for S_0_, see Figure S13). In this line, the coordination bond distances keep on shortening for the S_8_ state, although the coordination sphere is more distorted (S_T_ = 0.406 for S_8_). Based on these optimized geometries, the emission energies from both excited states could be calculated as their vertical excitations from their corresponding ground state geometries, i.e., by subtracting the ground state energies from those of the excited states, in such a way that 366 and 345 nm were calculated for the S_1_ and S_8_ excited states. On the basis of these results, taking into account the redshift existing for the calculated lines with respect to the experimental emission (with λ_em,max_ = 394 nm), it can be guessed that both excited states converge on the same emission line and that the observed experimental emission band derives from the HOMO ← LUMO electronic transition with LC character (Figure 7).

**Figure 7:**
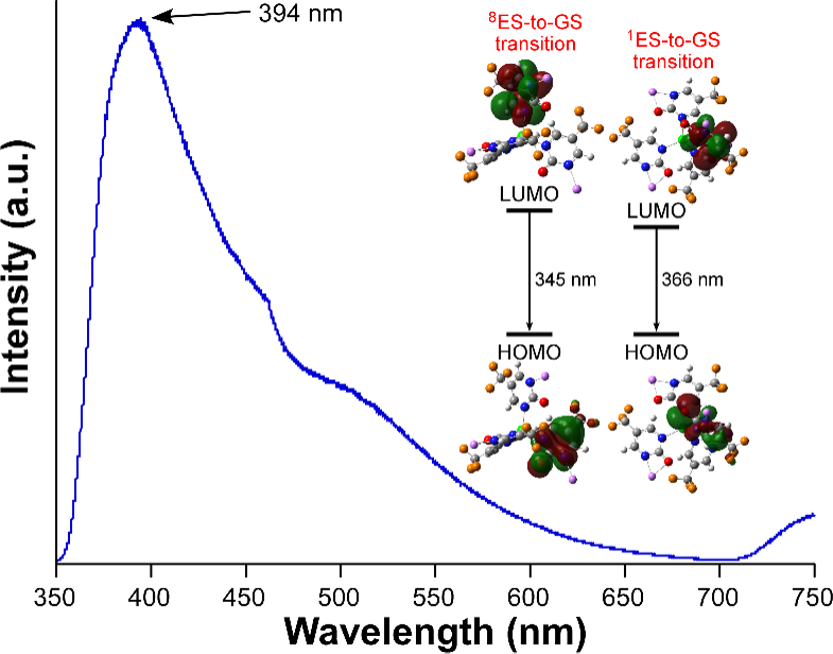
Room temperature emission spectrum of **GR-MOF-30** with its assignation.

The analysis of the emission lifetimes was performed on the basis of the fitting of the decay curves to the exponential expression [*I*_t_ = *A*_0_ + *A*_1_exp­(*t*/τ_1_) + *A*_2_exp­(*t*/τ_2_)] (where the required number of components varied according to the signal analyzed; see Table S7). This analysis reveals that, for both compounds, the first main band presents short-lived average components (in the range of 1 ns, see Table 1), of fluorescent nature, in concordance with the aforementioned ligand-centered excitations (see Figures S14 and S15). However, the lifetimes are substantially longer for the emission shoulders observed at 500 and 450 nm, respectively, for **GR-MOF-30** and **GR-MOF-31**, with values in the range of microseconds and thus in the range of phosphorescence. This fact seems to indicate that this high-wavelength zone of the spectrum (of lower intensity and hence shown as a shoulder of the main band) could be related to the emission arising from triplet states, as already observed for other similar compounds.^31–33^ To get deeper insights into that possibility, an additional TD-DFT calculation based on the optimized ground state (GS) geometry to locate the lowest-lying triplet excited states and estimate their relative energy with respect to GS was performed, from which we can conclude that the observed emission proceeds from the excited T_1_ state (see Table S6 and Figure S16).

**Table 1:** Selected Photophysical Data for Compounds **1** and **2**

comp.	*T* ^a^	λ_ex,max_ ^b^	λ_em,max_	τ_av_	QY (%)
**GR-MOF-30**	296	330^w^/350	394/500^sh^	0.94 ns/28.9 μs	5.2(4)
	25	304^w^/316^w^/329^w^/342	398/500^sh^	5.14 ns/48.1 ms	
**GR-MOF-31**	296	305^w^/340/(415)	388/450^sh^	1.12 ns/164.7 μs	6.5(5)
	25	335/(375)	374^sh^/385/415^sh^/450	2.81 ns/69.2 ms	

a Temperature given in K.

b Wavelengths are given in nm. Codes as superscripts: w = weak band, sh = shoulder. Those wavelengths given between brackets for compound **2** are only observed for the emission band at λ_em_ = 450 nm.

Taking into account that triplet state excitons are greatly quenched at room temperature due to the good overlapping of these energies with molecular vibrations, we decided to repeat the characterization of the samples at low temperatures. The excitation and emission spectra of **GR-MOF-30** do not substantially change with respect to RT, but for the fact that at 25 K, the main excitation band is slightly blue-shifted, and some weak bands peaking at lower wavelengths also emerge from the background. The steady-state spectrum of **GR-MOF-31** instead presents significant differences. On the one hand, upon laser excitation (λ_ex_ = 325 nm), a second intense band peaking at 450 nm is observed in addition to the main band with a maximum at 385 nm (see the light blue spectrum in Figure 6b, now showing shoulders at 374 and 415 nm), which could represent an enhanced emission of the compound’s phosphorescence. On the other hand, the second emission band (λ_em_ = 450 nm) seems to be, in fact, largely favored compared to the first emission band (λ_em_ = 385 nm) in view of the occurrence of a very intense excitation band peaking at 375 nm that is only observed when monitoring the second emission band (λ_em_ = 450 nm, not for the first band at λ_em_ = 385 nm, see green spectra in Figure 6b), while both bands share the excitation band already observed at RT (slightly blue-shifted to λ_ex_ = 335 nm at 25 K, see all data in Table 1). This fact means that, when the wavelength of the employed excitation light is slightly increased from 325 to 335 nm, thus better tuning to the first excitation band, the observed emission spectrum is entirely changed to be dominated by the band peaking at 450 nm (whereas the bands at λ_em_ = 415 and 385 nm are shown as shoulders with decreasing intensity based on the previous order, i.e., the band at λ_em_ = 385 nm showing the least intensity). It is worth noting that the emission spectrum recorded at λ_ex_ = 375 nm reflects a similar emission pattern with the main band peaking at λ_em_ = 480 nm (see Figure S17 and details described there). With all these changes in mind, emission decay curves were measured at the most relevant wavelengths to see the evolution of the processes with temperature (Table S7 and Figures S14 and S17). Starting from **GR-MOF-30**, it is observed that freezing the sample at 25 K brings significant enlargements of the liveness of the two processes, with the lifetime of the fluorescent signal (λ_em,max_ = 394 nm) being five times longer than at RT, and the phosphorescent emission (λ_em,max_ = 500 nm) being enlarged by 3 orders of magnitude. As a consequence, such a large phosphorescence falls within the long-lasting regime (τ > 20 ms) that makes the afterglow be traced by the human eye after the turn-off of the excitation source.^47^ This trend is similarly observed for **GR-MOF-31**, wherein both fluorescent and phosphorescent signals, with main λ_em,max_ = 385 and 450 nm (according to steady-state spectra recorded under λ_ex_ = 325 nm), experience enhancements of the same magnitude (see Table 1), among which the increase of the phosphorescence lifetime up to τ_av_ of ca. 70 ms is to be highlighted. At last, with the aim of better characterizing the phosphorescent emissions observed in both compounds, time-resolved emission spectra (TRES) were measured at 25 K (Figure 8). At first glance, the intensity of the phosphorescent band is much greater for **GR-MOF-31**, which is in good agreement with the fact that only a remarkable band could be observed for that compound in the steady-state spectrum. It is also observed that the decay is very gradual for this material, since even after 5 ms, the intensity of the band maximum is greater than the intensity of **GR-MOF-30** at 1 ms. Furthermore, for **GR-MOF-31**, it is also observed that the band related to fluorescence (388 nm) completely vanished after 5 ms. These facts are also reflected in the visual emission of the phosphorescent signal of both compounds, perceived as a slightly longer afterglow in the case of **GR-MOF-31**, as observed in the photographs (see the insets in Figure 8), from which long-lasting phosphorescence is inferred.

**Figure 8:**
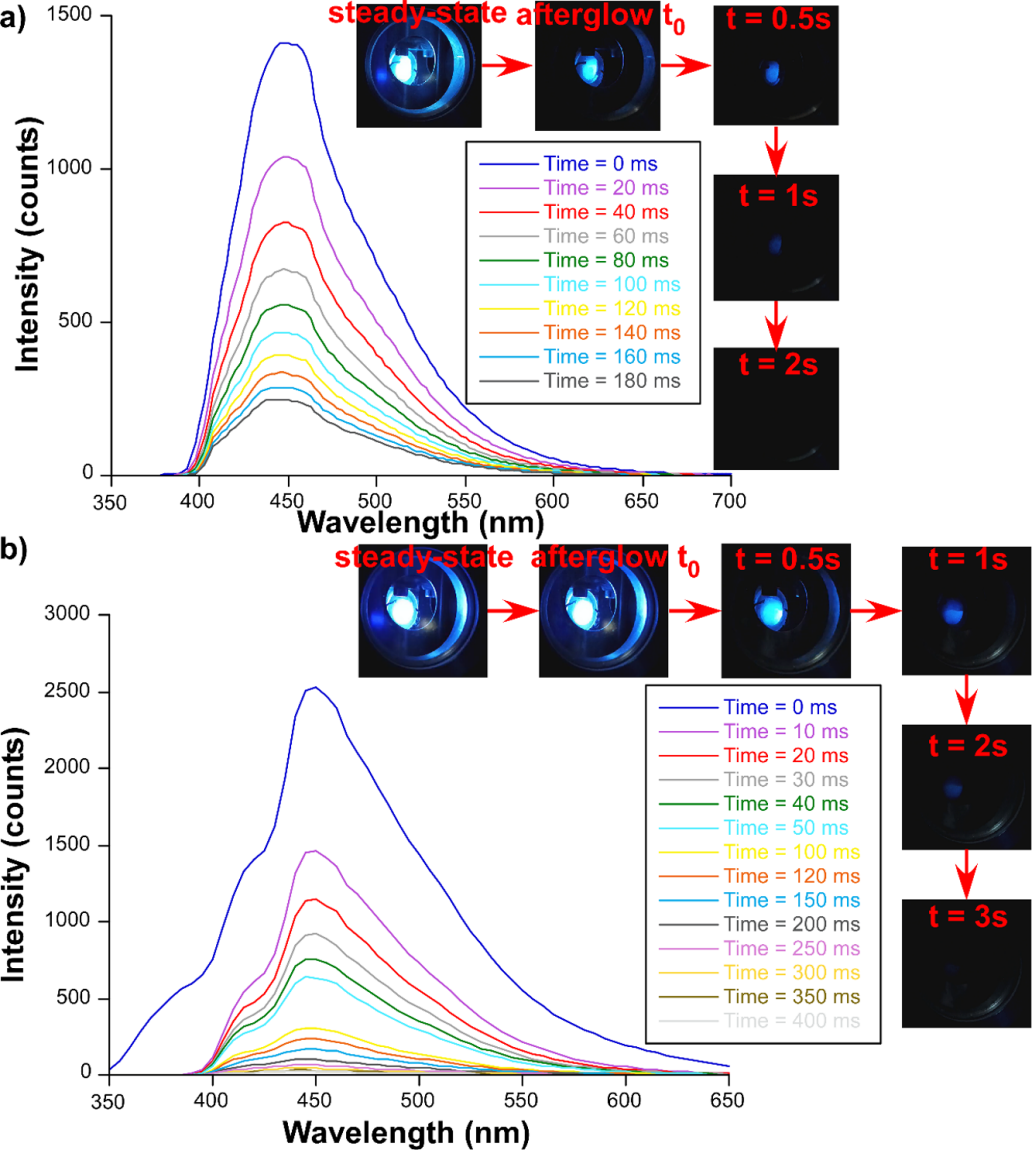
TRES of **GR-MOF-30** and **GR-MOF-31** recorded at 25 K showing selected decays (λ_ex_ = 340 nm). Insets show photographs taken at variable times elapsed after the initial excitation when the irradiation turns off.

In addition to temperature, other environmental factors such as the presence of air were also explored. In a simple experiment, the emission spectra of both samples were repeated at RT but under open atmospheric conditions, i.e., in the absence of vacuum. As observed in Figure S18, the emission profiles of both samples remain almost unchanged, although some slight but remarkable changes occur for **GR-MOF-30**. On the one hand, the band maximum presents a slight red shift when the sample is placed under high vacuum compared to the open atmospheric conditions. On the other hand, the region covering 450–600 nm, associated with the phosphorescent emission of the sample, experiences an increase in emitted intensity. These two effects, happening only in **GR-MOF-30**, could be related to the greater flexibility of its framework compared to that of **GR-MOF-31**, wherein CF_3_···aromatic ring interactions take place among neighboring ligand molecules.

Finally, to end up with the characterization of photoluminescence properties, the emission photostability under continuous irradiation was evaluated, given that coordination polymers may suffer from degradation owing to intense vibrations occurring during light absorption in the bulk state,^48,49^ meaning this is an important parameter to be considered for the real implementation of the material in a specific application. In fact, some brightly and efficiently emitting lanthanide-based compounds, such as [Tb­(acac)_3_(H_2_O)_3_] (acac = acetylacetonate),^50^ are known to lose 53% of their initial intensity after 6 h of continuous illumination, whereas other materials such as the coordination polymer of [Ru­(dqpCOO)­Zn­(OOCH)_2_]_*n*_ (with dqp = 2,6-di­(quinolin-8-yl)-pyridine carboxylic acid and OOCH = formic acid)^51^ present almost no degradation being exposed to halogen lamps of similar power. For the present experiments, we employed He–Cd laser light to test the stability of the samples owing to its high irradiance of 54 mW cm^–2^ (see further details in the Experimental Section), measuring the emitted intensity of their corresponding band maxima (at λ_em_ = 392 and 384 nm) during 5 h. As observed in Figure 9, both samples present substantial photodegradation under these harsh conditions, with **GR-MOF-31** showing remarkably higher stability than **GR-MOF-30** (reaching losses of 55 and 34% for **GR-MOF-30** and **GR-MOF-31**, respectively). Moreover, it is also worth noting that the decaying profile of **GR-MOF-31** presents a plateau from 200 min on, meaning that it could be showing a quite stable signal for longer exposure times. Nevertheless, the structural stability of both compounds was kept after the irradiation, as evidenced by PXRD (Figures S19 and S20). These results are also in line with the general trends of higher physicochemical stability observed for **GR-MOF-31**, meaning that its stronger supramolecular structure, benefiting from CF_3_···aromatic ring dispersive contacts, could be decisive for the durability of the compound.

**Figure 9:**
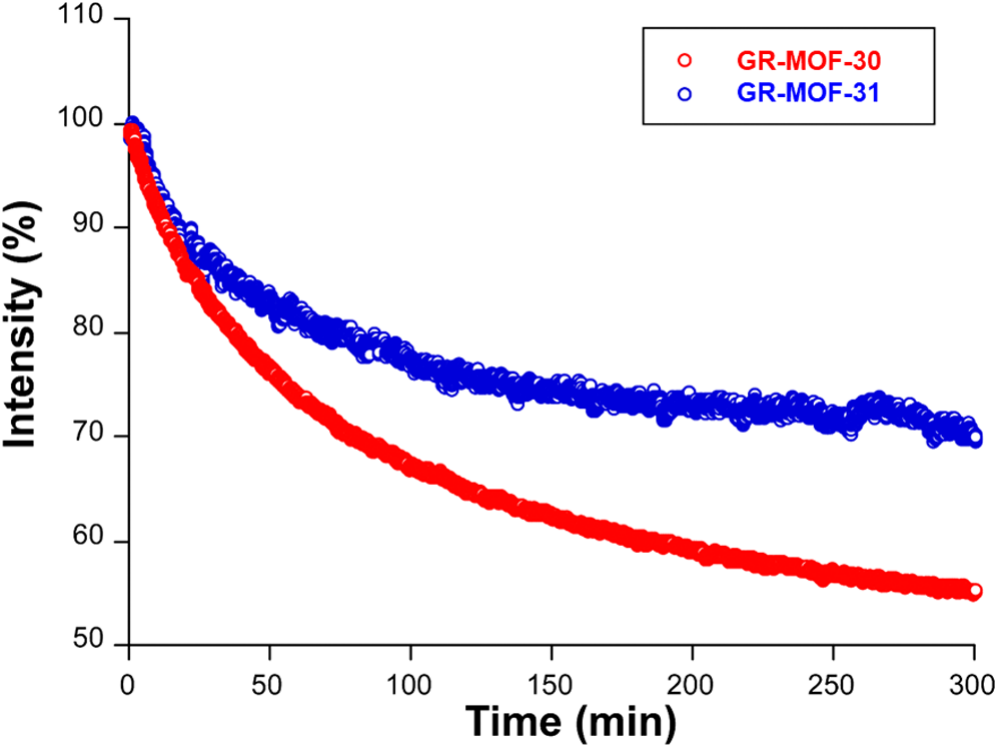
Photostability results in the form of intensity vs time plots for **GR-MOF-30** and **GR-MOF-31** recorded at room temperature under laser UV irradiation (λ_ex_ = 325 nm).

## Conclusions

In this work, two novel 3D-MOFs based on group 12 transition metals and a ligand derived from an S_N_Ar reaction have been synthesized. Both compounds present a compact architecture without porosity, which allows optimal interaction between neighboring ligands, thus enhancing the luminescent properties of the materials. It is necessary to highlight the stacking interactions (CF-π) present in **GR-MOF-31**, which are not found in **GR-MOF-30**. This type of interaction not only brings slightly higher thermal stability but also seems to be related to their different luminescent behavior. Solid-state photoluminescence measurements show that both materials provide interesting luminescence behavior. **GR-MOF-30** shows a fluorescence maximum at 394 nm and a phosphorescent maximum around 500 nm with a maximum lifetime of 48.1 ms, whereas **GR-MOF-31** presents remarkable long-lasting phosphorescence, with a lifetime of 69.2 ms. Besides, the emission of **GR-MOF-31** seems to be more influenced by temperature compared to **GR-MOF-30**. This is observed when its spectra are measured at low temperatures, where the emission of **GR-MOF-30** barely varies with the spectrum at room temperature, while that for **GR-MOF-31** goes from dominating a fluorescent emission (RT) to a phosphorescent emission (25 K). A priori, such a difference may facilitate an application route for the material as a temperature sensor, where more studies are required to accurately analyze this type of behavior. Instead, the higher cohesion of the supramolecular structure of **GR-MOF-31** (CF-π interactions) compared to **GR-MOF-30** imbues the former with more stable luminescence against other environmental factors such as the presence of air, in addition to the fact that **GR-MOF-31** presents higher photostability with a low signal intensity loss of only 34% when exposed to a beam with high irradiance (54 mW cm^–2^).

## Supplementary Material



## Notes

Crystallographic refinement details, reaction mechanism, thermogravimetric analysis, detailed structural images, powder X-ray diffraction patterns, diffuse reflectance spectra, fluorescence emission spectra, emission decay curves, and simulation of both compounds (PDF)
