# Supplementary material for: In Situ Ligand Transformation for the Development of Luminescent 3D Metal–Organic Frameworks with Diamond-like Topology
Source: Cryst Growth Des. 2025 Jan 22;25(3):703–12. doi: 10.1021/acs.cgd.4c01496 (PMC12150766; doi:10.1021/acs.cgd.4c01496)
Supplement: Supplementary file 1 [file cg4c01496_si_001.pdf]

In-situ Ligand Transformation for the Development of Luminescent 3DMetal-Organic Frameworks with Diamond-like Topology

Antonio A. García-Valdivia,[a] Sara Rojas,[a] Duane Choquesillo-Lazarte,[b] Antonio Rodríguez-Diéguez,[a] José Ángel García,[c] Javier Cepeda,*[d] Pablo Salcedo-Abraira *[a]

[a] Departamento de Química Inorgánica, Facultad de Ciencias, Universidad de Granada, 18071 Granada, Spain.
[b] Laboratory for Crystallographic Studies IACT, CSIC-UGR, Av. Las Palmeras nº4, 18100 Granada, Spain.
[c] Departamento de Física, Facultad de Ciencia y Tecnología, Universidad del País Vasco/Euskal Herriko Unibertsitatea (UPV/EHU), 48940, Leioa, Spain.
[d] Departamento de Química Aplicada, Facultad de Química, Universidad del País Vasco (UPV/EHU), Paseo Manuel Lardizábal 3, 20018 Donostia-San Sebastián, Spain.

Table S1. Crystallographic Data and Structural Refinement Details for **GR-MOF-30** and **GR-MOF-31**.

**Compound**	**GR-MOF-30**	**GR-MOF-31**
Formula	$C_{10}H_4F_6N_4O_2Zn$	$C_{10}H_4F_6N_4O_2Cd$
Formula weight	391.54	438.57
CCDC	2388248	2388249
Temperature/K	100(2)	100(2)
Crystal system	Tetragonal	Orthorhombic
Space group	$I\bar{4}2d$	$Pna2_1$
a/Å	8.9635(4)	9.8919(7)
b/Å	8.9635(4)	12.3580(9)
c/Å	15.0012(6)	10.1629(7)
α/°	90	90
β/°	90	90
γ/°	90	90
Volume/Å^3	1205.26(1)	1242.35(2)
Z	4	4
ρ_{calc}/ g/cm^3	2.158	2.345
μ/mm^{-1}	2.134	1.851
F(000)	768.0	840.0
Crystal size/mm^3	0.12 × 0.1 × 0.1	0.12 × 0.1 × 0.09
Radiation	MoKα (λ = 0.71073)	MoKα (λ = 0.71073)
2Θ range for data collection/°	5.294 to 55.012	5.19 to 55.096
Index ranges	$-11 \leq h \leq 11$, $-11 \leq k \leq 11$, $-19 \leq l \leq 19$	$-12 \leq h \leq 11$, $-16 \leq k \leq 16$, $-13 \leq l \leq 13$
Reflections collected	6052	26476

Independent reflections	702 [R_{int} = 0.0540, R_{sigma} = 0.0285]	2862 [R_{int} = 0.0393, R_{sigma} = 0.0218]
Data/restraints/parameters	702/37/64	2862/1/208
Goodness-of-fit on F^2	1.202	1.093
Final R indexes [I>=2σ (I)]	R_1 = 0.0484, wR_2 = 0.1218	R_1 = 0.0158, wR_2 = 0.0395
Final R indexes [all data]	R_1 = 0.0515, wR_2 = 0.1243	R_1 = 0.0159, wR_2 = 0.0396
Largest diff. peak/hole / e $Å^{-3}$	0.72/-0.74	0.46/-0.42
Flack parameter	0.019(17)	-0.003(7)

[a]R_1 = S||F_o| - |F_c||/S|F_o|. [b]Values in parentheses for reflections with I > 2s(I). [c]wR_2 = {S[$w(F_o^2 - F_c^2)^2$] / S[$w(F_o^2)^2$]}^{½}

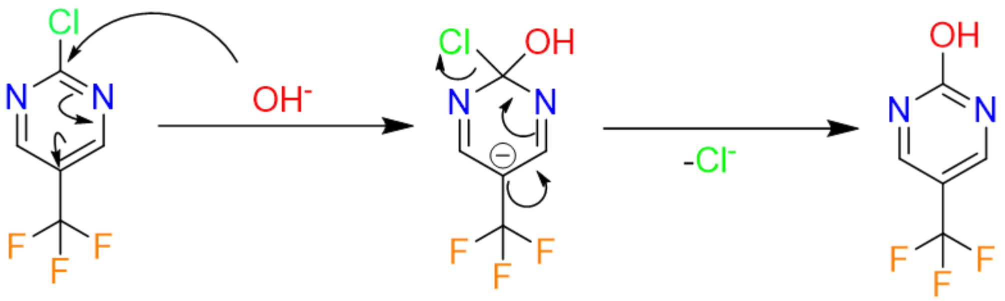

Scheme S1. S_NAr mechanism proposed for the *in situ* 2-hydroxy-5-(trifluoromethyl)pyrimidine ligand formation.

Table S2. Selected bond lengths (Å) and angles (º) for **GR-MOF-30**.[a]

Atom	Atom	Length(Å)	Atom	Atom	Atom	Angle(˚)
Zn1	$N1^1$	2.020(5)	$N1^1$	Zn1	$N1^2$	107.87(15)
Zn1	$N1^2$	2.020(5)	$N1^2$	Zn1	N1	112.7(3)
Zn1	N1	2.020(5)	$N1^1$	Zn1	N1	107.87(15)
Zn1	$N1^3$	2.020(5)	$N1^1$	Zn1	$N1^3$	112.7(3)
			$N1^2$	Zn1	$N1^3$	107.87(15)
			$N1^3$	Zn1	N1	107.88(15)

[a]Symmetry operations (1)+y,1-x,1-z; (2)1-y,+x,1-z; (3)1-x,1-y,+z

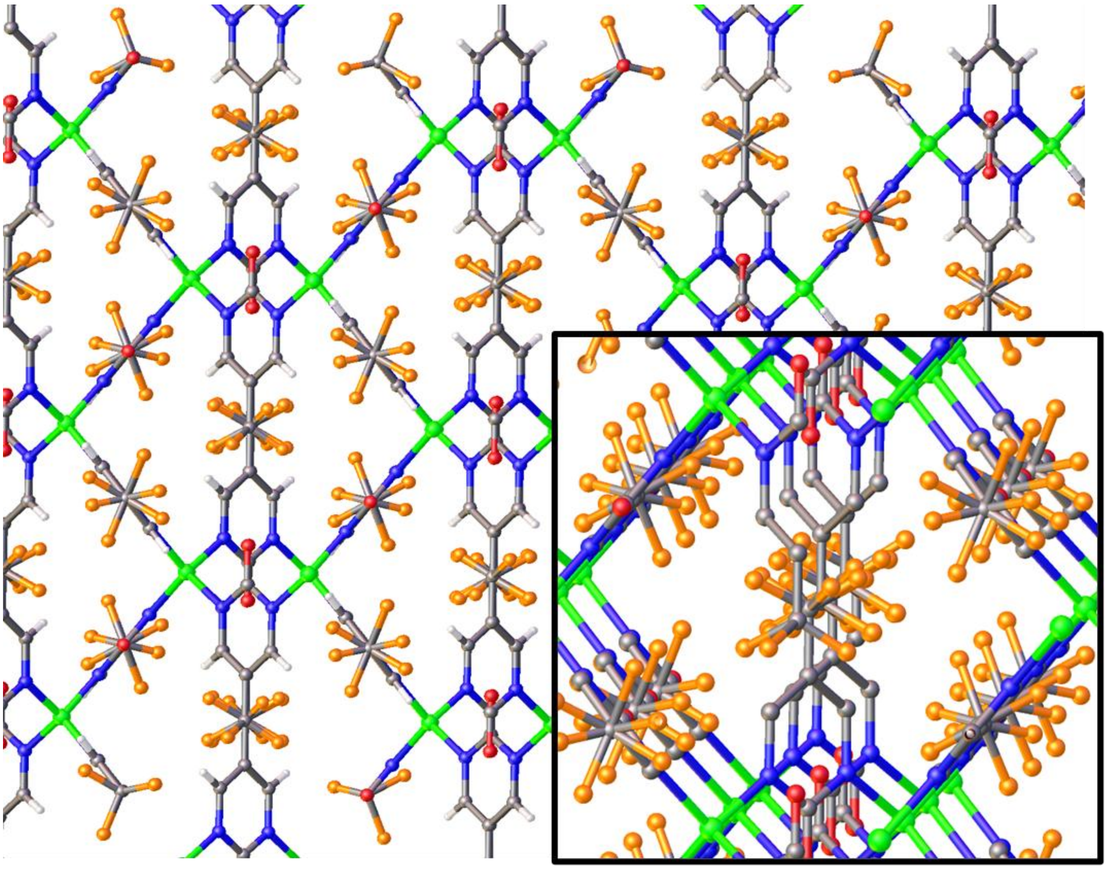

Figure S1. View along *a* axis of **GR-MOF-30**. Inset: View of obstructed channels. H atoms were removed in terms of clarity in the inset.

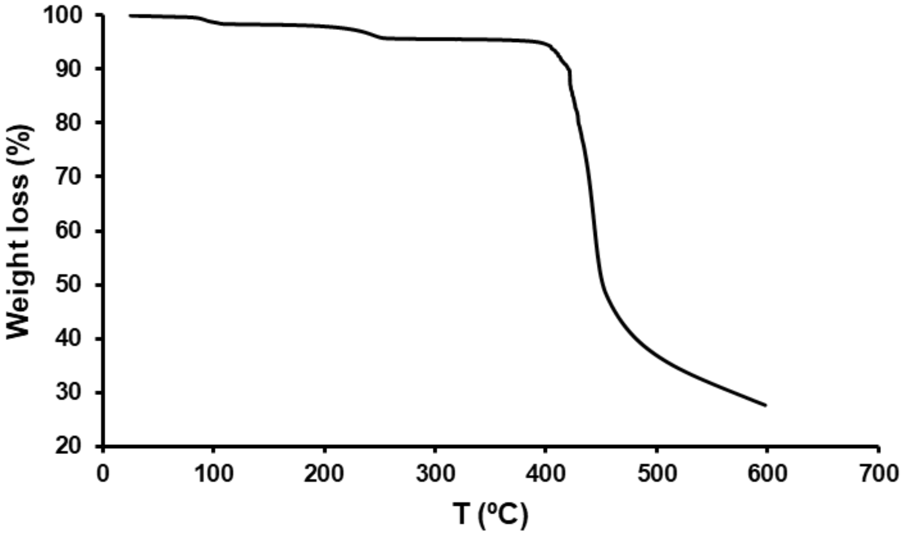

Figure S2. TGA curve of **GR-MOF-30.**

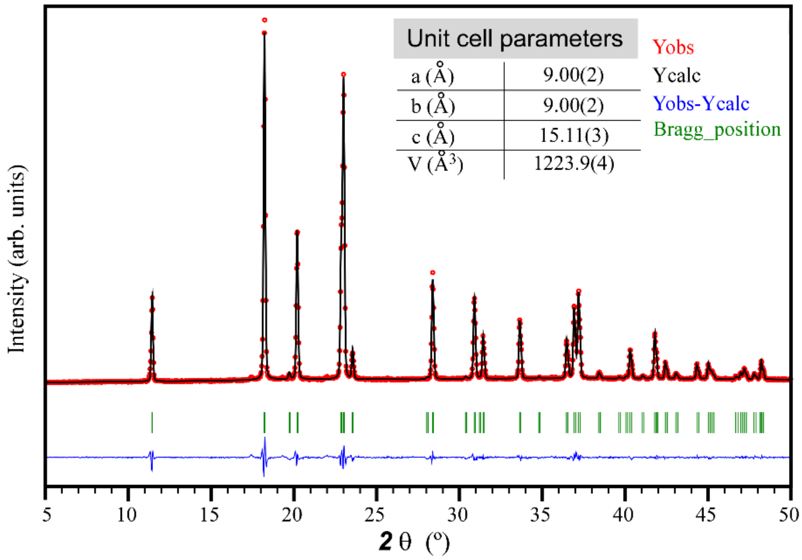

Figure S3. Pawley profile fitting of **GR-MOF-30.**

Table S3. Comparison between the unit cell parameters obtained by SC-XRD and PXRD for **GR-MOF-30** and **GR-MOF-31**.

Compound	**GR-MOF-30**		**GR-MOF-31**	
Technique	SC-XRD	PXRD	SC-XRD	PXRD
Space Group	$I\bar{4}2d$	$I\bar{4}2d$	$Pna2_1$	$Pna2_1$
a/Å	8.9635(4)	9.00(2)	9.8919(7)	9.95(3)
b/Å	8.9635(4)	9.00(2)	12.3580(9)	12.59(2)
c/Å	15.0012(6)	15.11(3)	10.1629(7)	10.18(2)
Volume/Å^3	1205.26(1)	1223.9(4)	1242.35(2)	1275.3(6)

Table S4. CShMs for the coordination environment of **GR-MOF-30** and **GR-MOF-31.**
Codes:

SP-4	1 D_{4h}	Square
T-4	2 T_d	Tetrahedron
SS-4	3 C_{2v}	Seesaw
vTBPY-4	4 C_{3v}	Vacant trigonal bipyramid

**Structure [ML_4]**	**SP-4**	**T-4**	**SS-4**	**vTBPY-4**
**GR-MOF-30**	30.686	**0.081**	8.700	3.650
**GR-MOF-31**	28.739	**0.891**	7.784	2.165

Table S5. Selected bond lengths (Å) and angles (°) for compound **GR-MOF-31.**[a]

**Atom**	**Atom**	**Length/Å**	**Atom**	**Atom**	**Atom**	**Angle/°**
Cd1	N1	2.237(2)	N1	Cd1	N2[1]	96.66(8)
Cd1	N2[1]	2.299(2)	N1	Cd1	N3	107.37(9)
Cd1	N3	2.250(2)	N1	Cd1	N4	117.28(8)

Cd1	N4	2.256(2)	N3	Cd1	N2[1]	111.95(8)
			N3	Cd1	N4	120.68(8)
			N4	Cd1	N2[1]	99.89(8)

[a]Symmetry operations (1)1/2+x,3/2-y

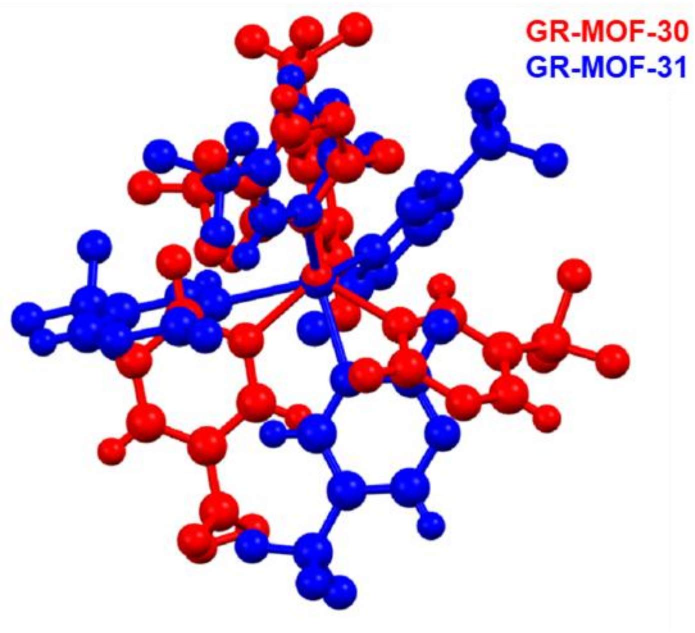

Figure S4. Structure overlay of the $[ML_4]^{2-}$ coordination excerpts of **GR-MOF-30** and **GR-MOF-31** to represent the relative geometrical differences in the building units.

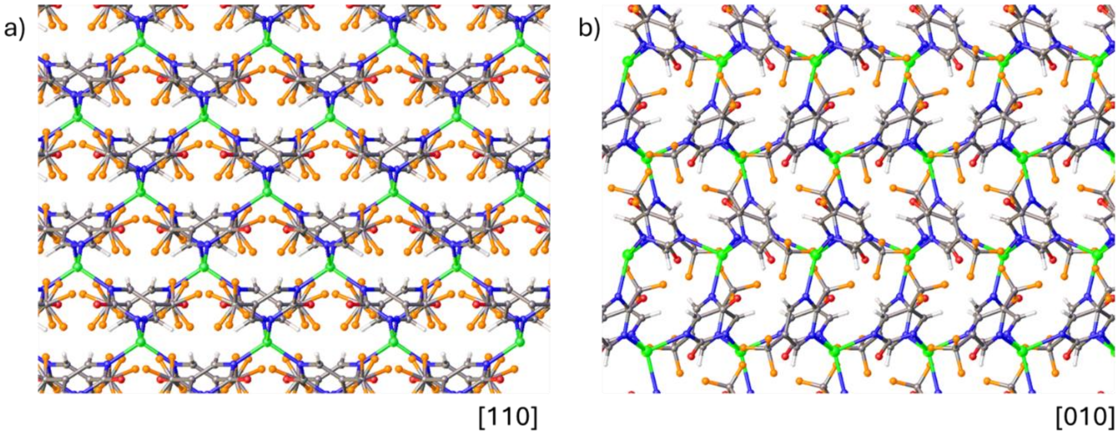

Figure S5. **GR-MOF-30** (a) **GR-MOF-31** (b) structures from different directions ([110] and [010], respectively) showing the rows of tetrahedra.

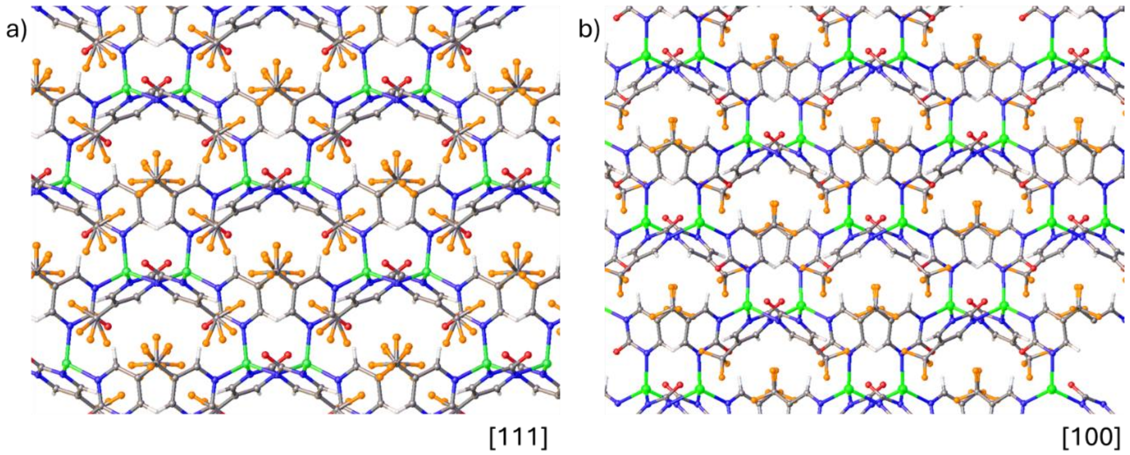

Figure S6. **GR-MOF-30** (a) and **GR-MOF-31** (b) structures from different directions ([111] and [100], respectively).

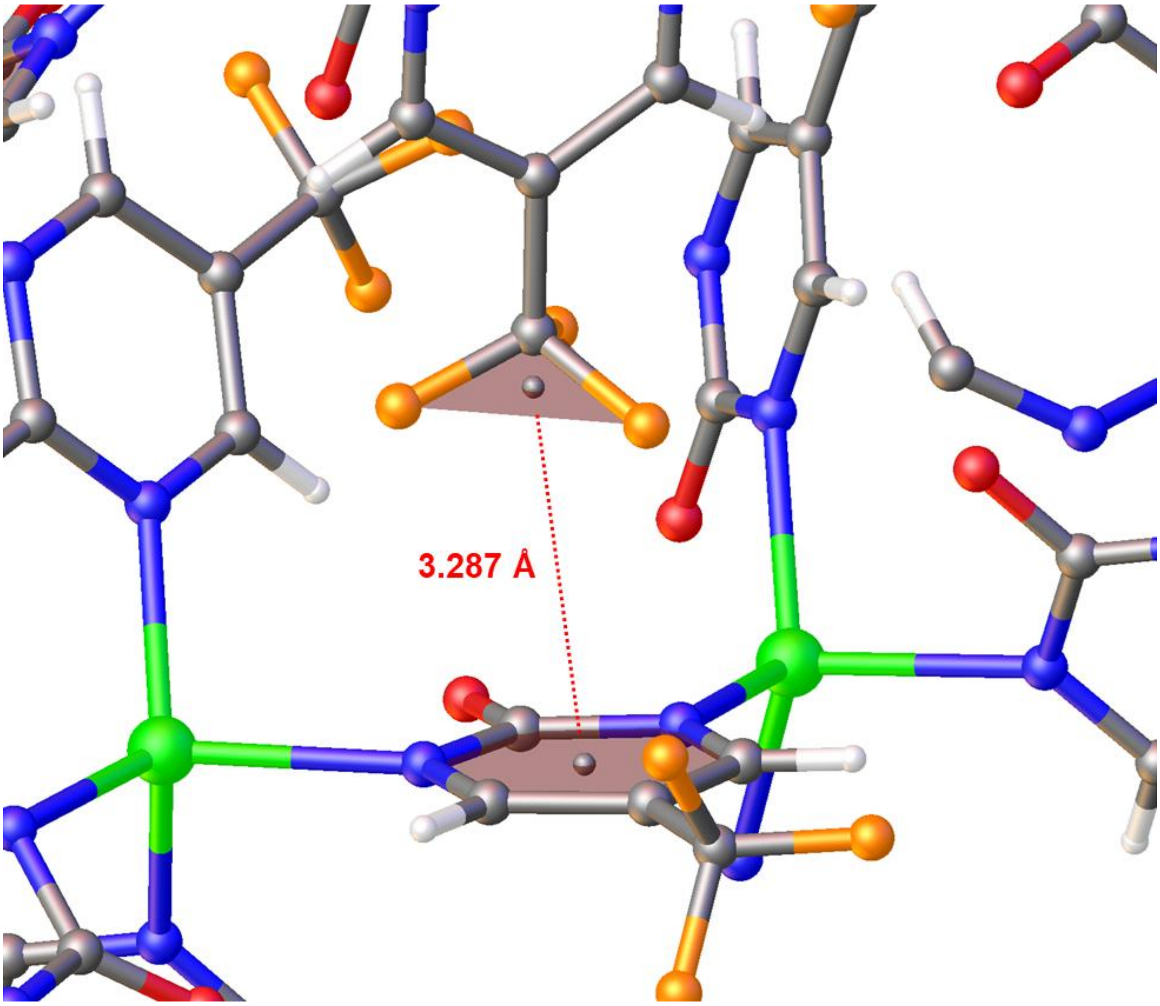

Figure S7. Potential interaction between centroids of CF_3 group and the aromatic ring of the ligand present in **GR-MOF-31**.

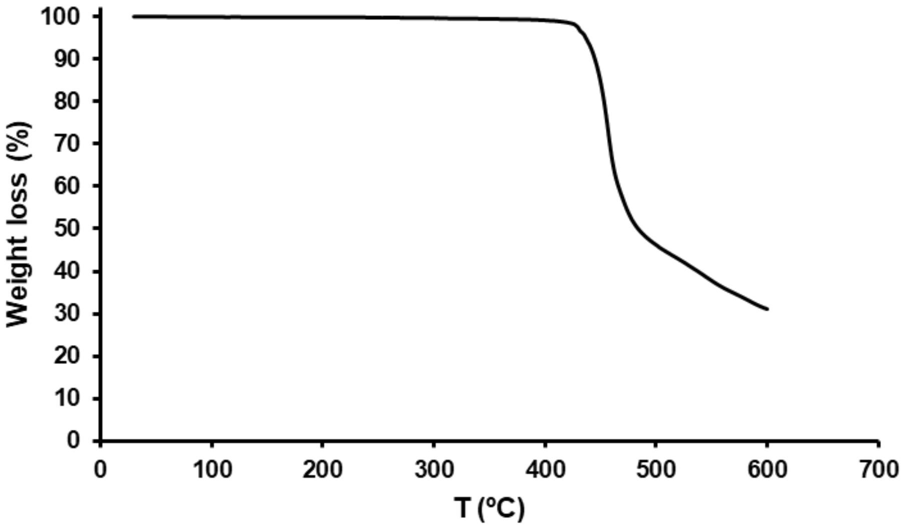

Figure S8. TGA curve of **GR-MOF-31**.

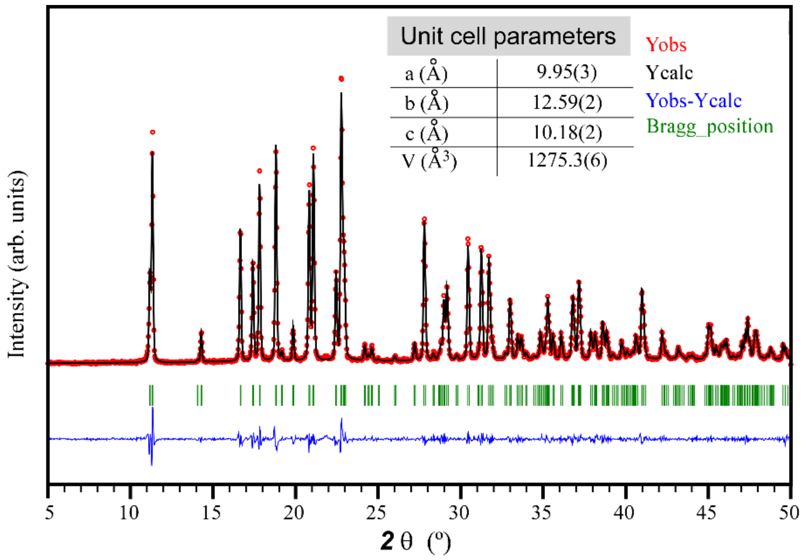

Figure S9. Pawley profile fitting of **GR-MOF-31**.

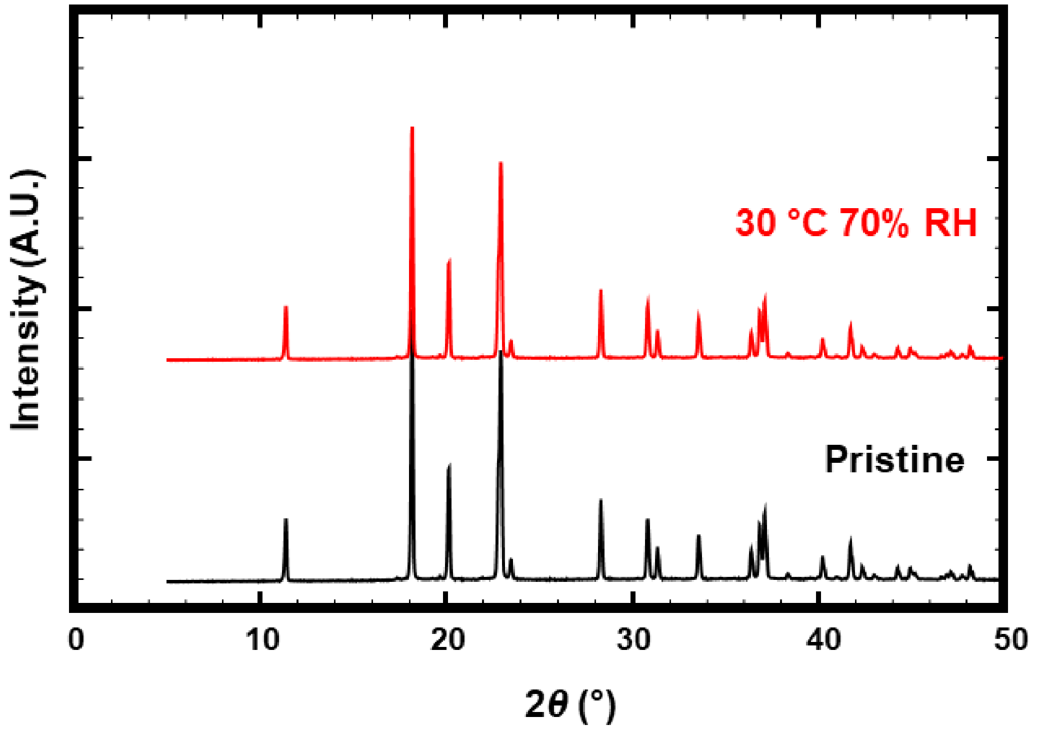

Figure S10. PXRD patterns of **GR-MOF-30** before (black) and after (red) the exposure at 70% RH at 30 °C during 24 h.

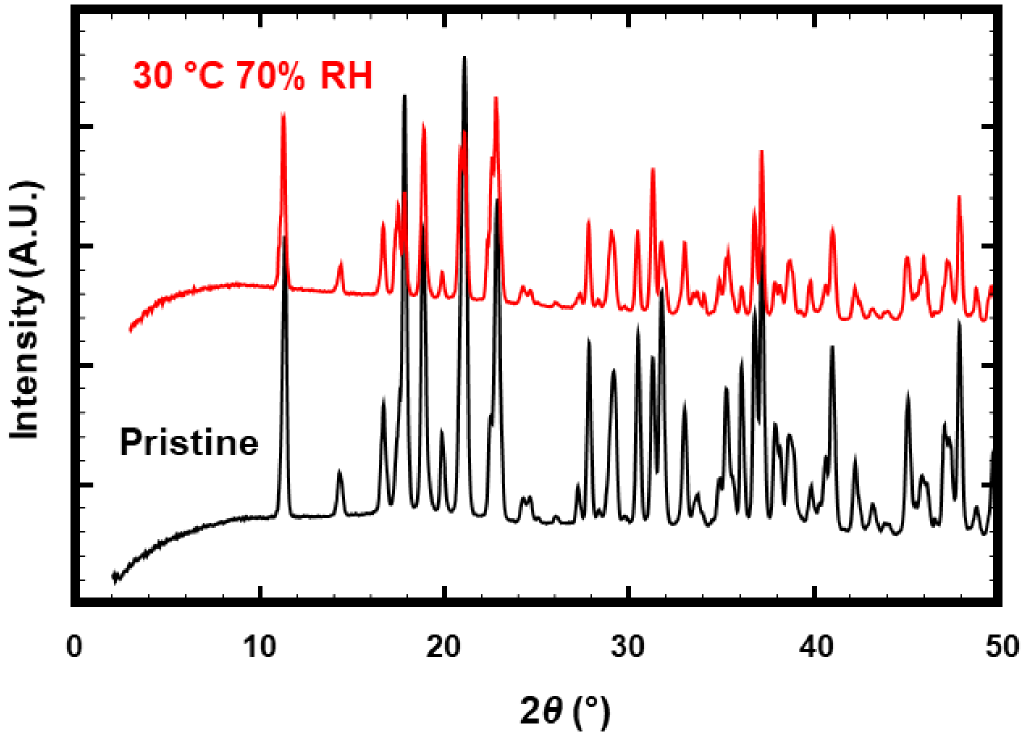

Figure S11. PXRD patterns of **GR-MOF-31** before (black) and after (red) the exposure at 70% RH at 30 °C during 24 h.

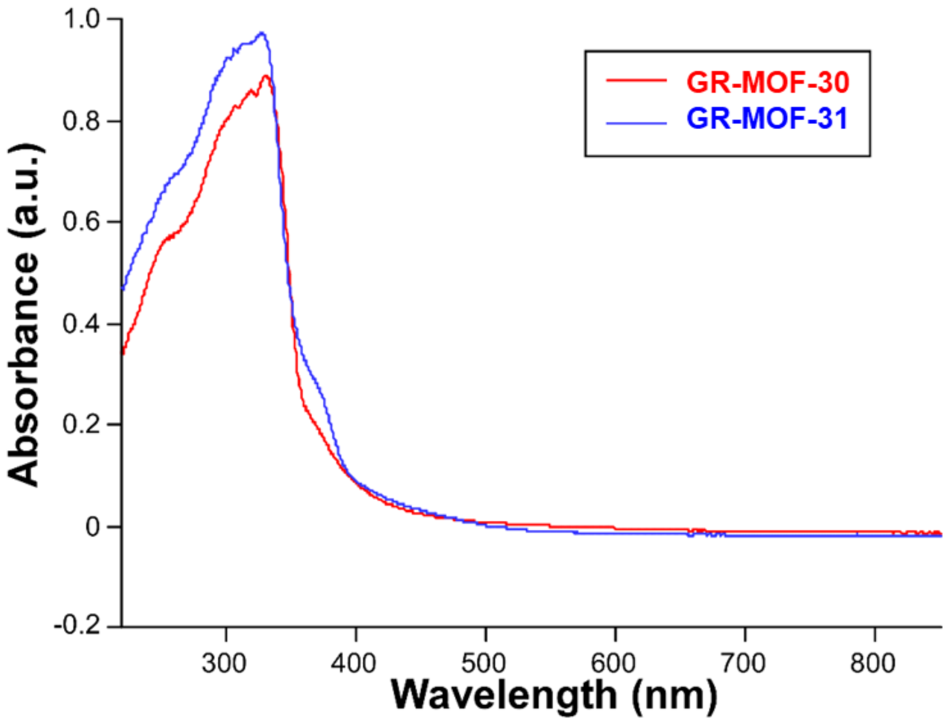

Figure S12. Diffuse reflectance spectra of **GR-MOF-30** and **GR-MOF-31**.

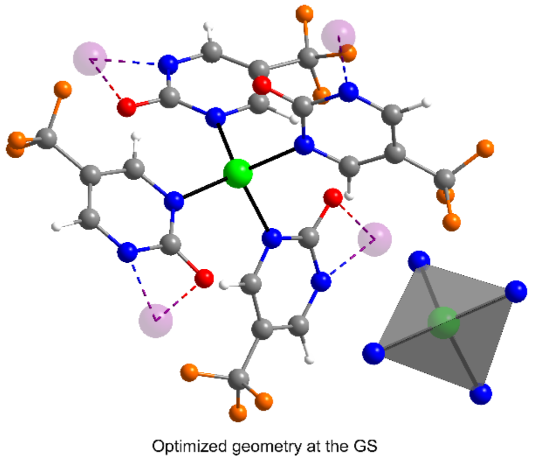

(a)

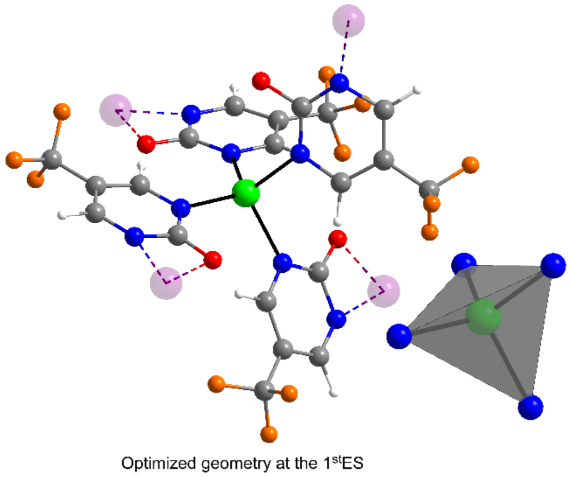

(b)

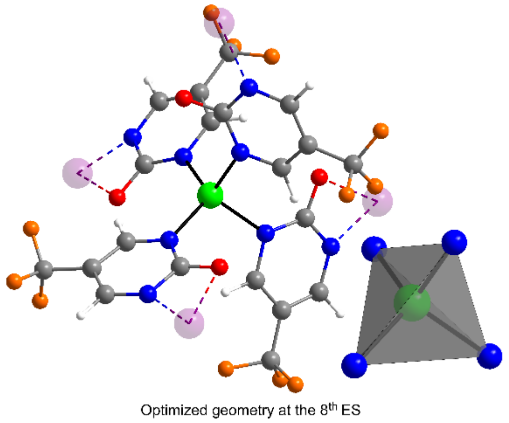

(c)

Figure S13. Optimized fragments of **GR-MOF-30** at: **(a)** S_0, **(b)** S_1 and **(c)** S_8 electronic states.

Table S6. Experimental (diffuse reflectance) and calculated main excitation wavelengths (nm). Singlet electronic transitions and associated oscillator strengths were calculated using a model of **GR-MOF-30** in gas phase.

Exp. λ	Calc λ	Significant contributions	State (S_n)	Osc. st. (a.u.)
332	316	HOMO → LUMO (73%)<br>HOMO → LUMO +1 (19%)	1	0.0786
300	298	HOMO – 3 → LUMO + 1 (42%)<br>HOMO – 3 → LUMO + 2 (18%)	8	0.0741
255	232	HOMO – 2 → LUMO + 8 (20%)<br>HOMO – 1 → LUMO + 8 (12%)<br>HOMO – 7 → LUMO + 3 (11%)	65	0.2209
**Exp. λ**	**Calc λ**	**Significant contributions**	**State (T_n)**	**Osc. st. (a.u.)**
500	485	HOMO → LUMO (95%)	1	0.0367

Table S7. Best fit results of decay curves performed at room temperatures monitoring different emission wavelengths for **GR-MOF-30** and **GR-MOF-31**.

**Fluorescence**					
**Compound**	**$\lambda_{ex/em}$ (nm)**	**Temp. (K)**	**τ_1 (ns) / weight (%)**	**τ_2 (ns) / weight (%)**	**Chi Sq.**
**GR-MOF-30**	340/394	296	0.123(5) / 53	1.86(2) / 47	1.357
	340/394	25	0.043(5) / 13	5.91(2) / 87	1.251
**GR-MOF-31**	340/388	296	1.121(4) / 100	-	1.276
	340/388	25	0.306(3) / 52	5.53(4) / 48	1.558

**Phosphorescence**						
**Compound**	**$\lambda_{ex/em}$ (nm)**	**Temp. (K)**	**τ_1 (μs) / weight (%)**	**τ_2 (μs) / weight (%)**	**τ_3 (μs) / weight (%)**	**Chi Sq.**
**GR-MOF-30**	340/500	296	7.1(1) / 57	57.9(9) / 43	-	1.187
	340/500	25	2018(180) / 3	16362(1333) / 22	60005(3060) / 74	1.321
**GR-MOF-31**	340/450	296	34.5(9) / 17	86.4(1) / 60	465(9) / 23	1.114

	340/450	25	17202(368) / 17	51819(763) / 58	144926(1982) / 25	1.428

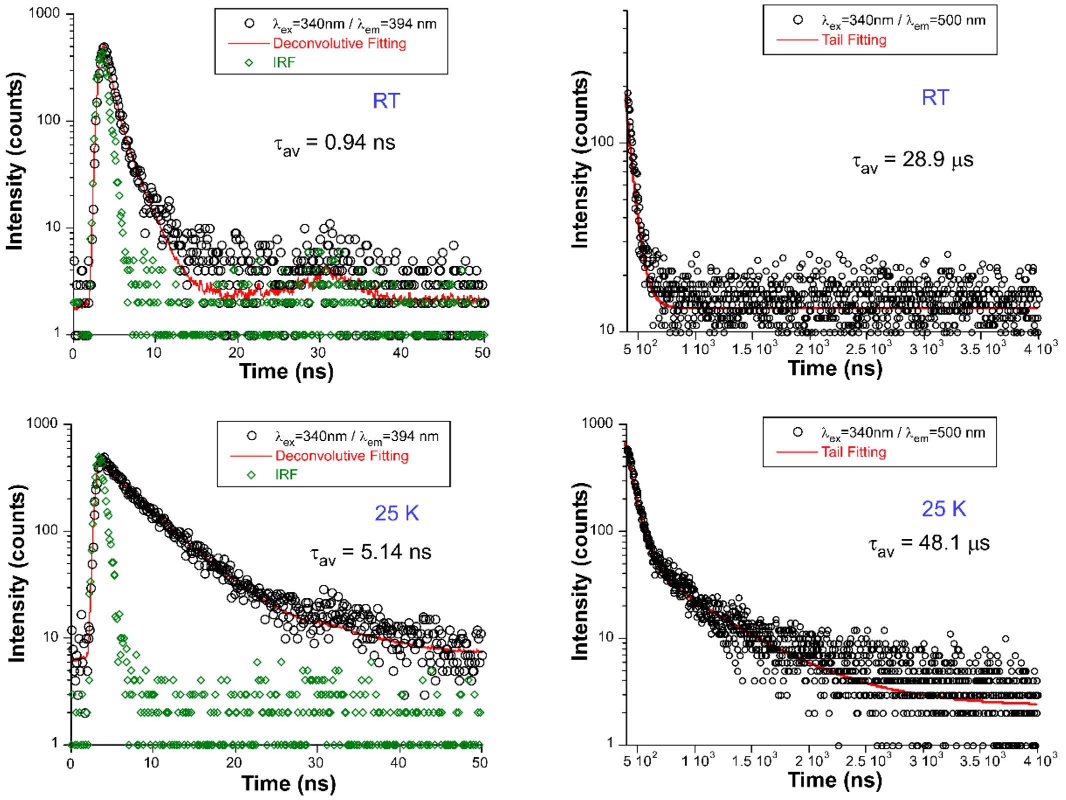

Figure S14. Emission decay curves with the best fittings to estimate the lifetimes of **GR-MOF-30** at variable temperature.

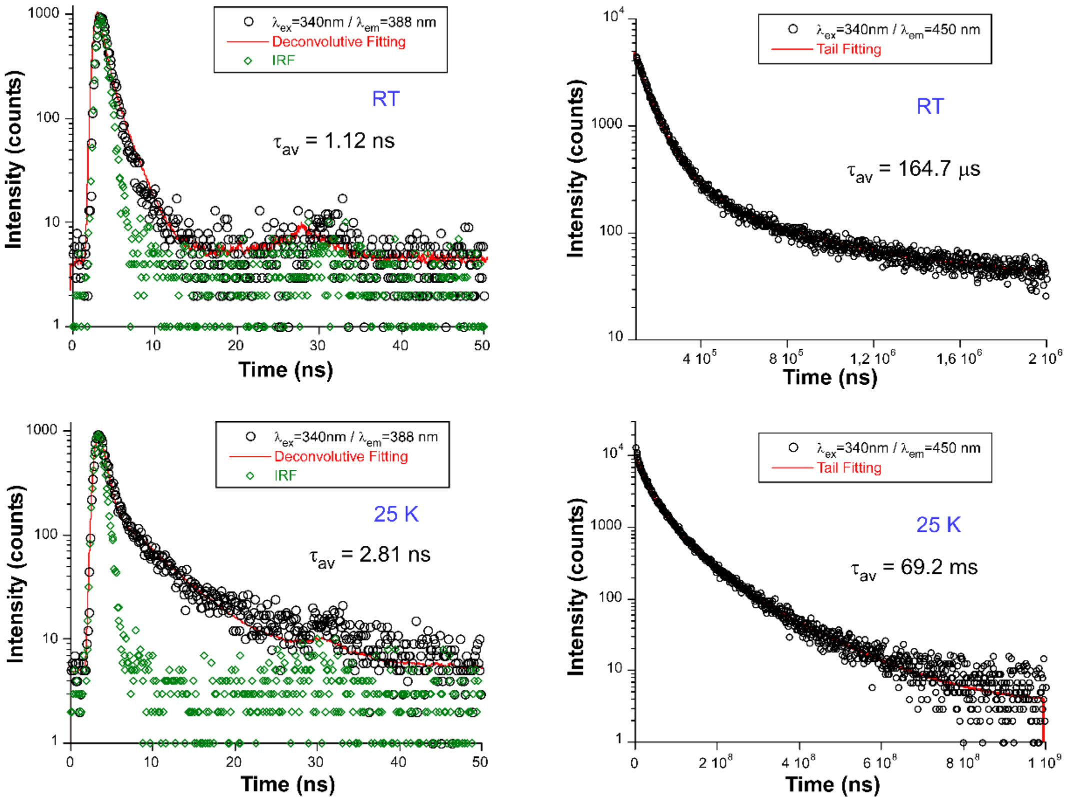

Figure S15. Emission decay curves with the best fittings to estimate the lifetimes of **GR-MOF-31** at variable temperature.

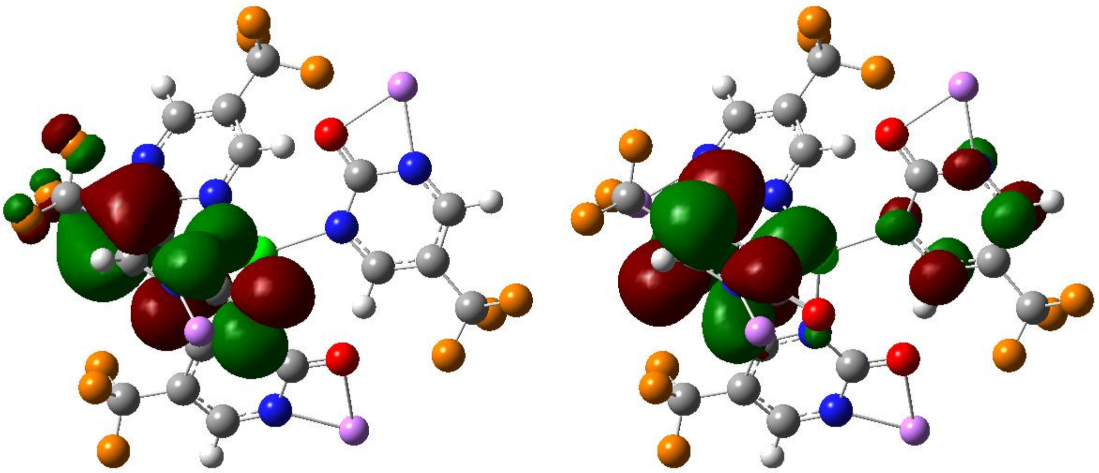

HOMO LUMO

Figure S16. Frontier molecular orbitals for the T_1 state of the optimized model of **GR-MOF-30**.

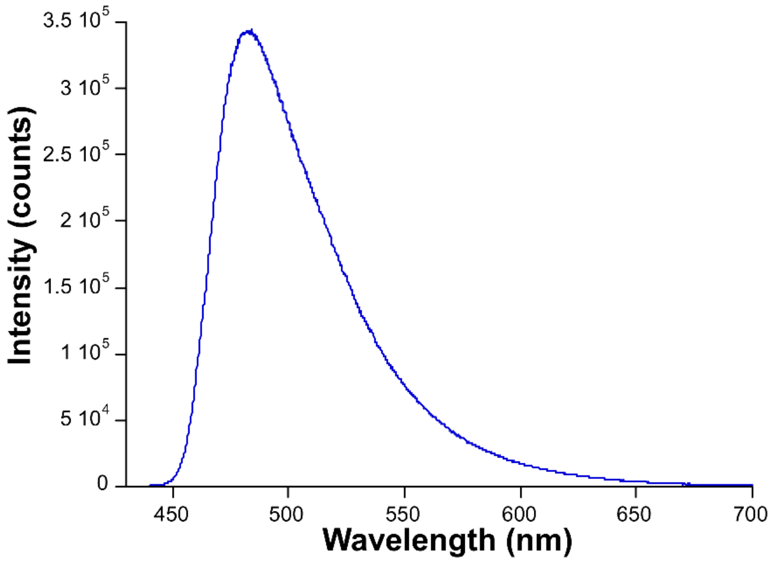

Figure S17. Emission spectrum of **GR-MOF-31** recorded at 25 K under λ_{ex} = 375 nm.

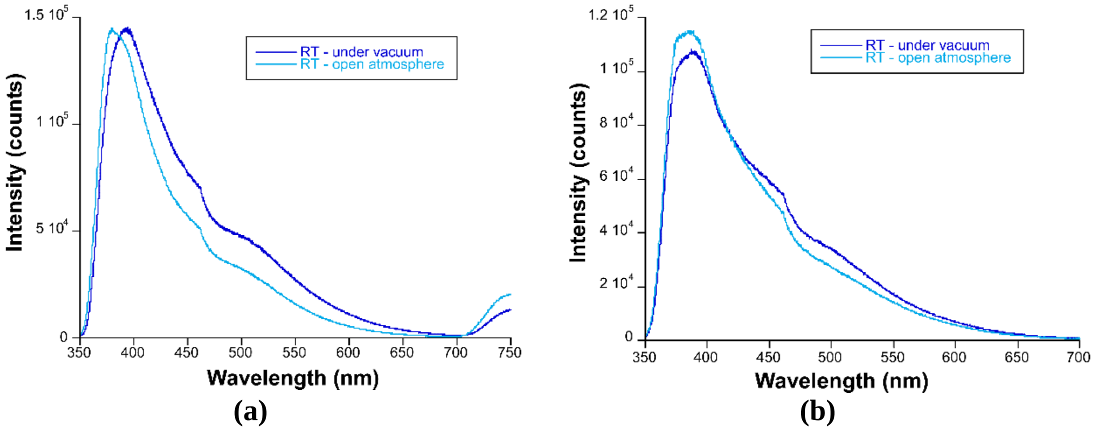

Figure S18. Comparative emission spectra at variable atmospheric conditions for samples of **GR-MOF-30 (a)** and **GR-MOF-31 (b)**.

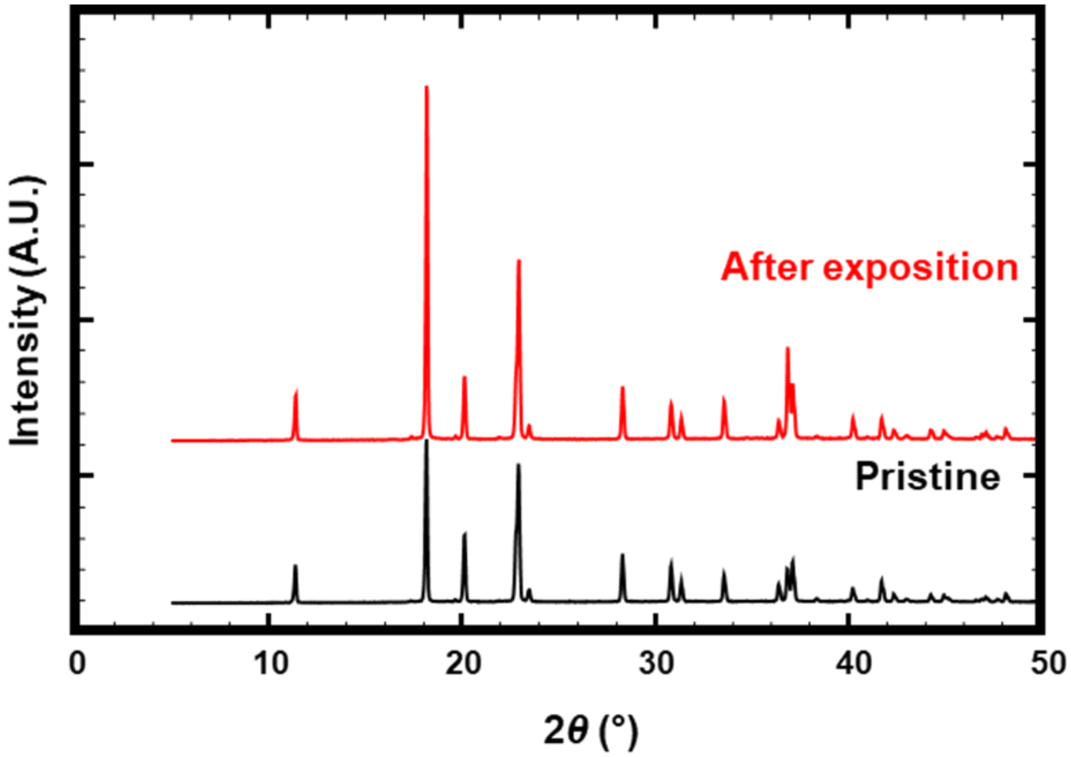

Figure S19. PXRD patterns of **GR-MOF-30** before (black) and after(red) of the photostability studies.

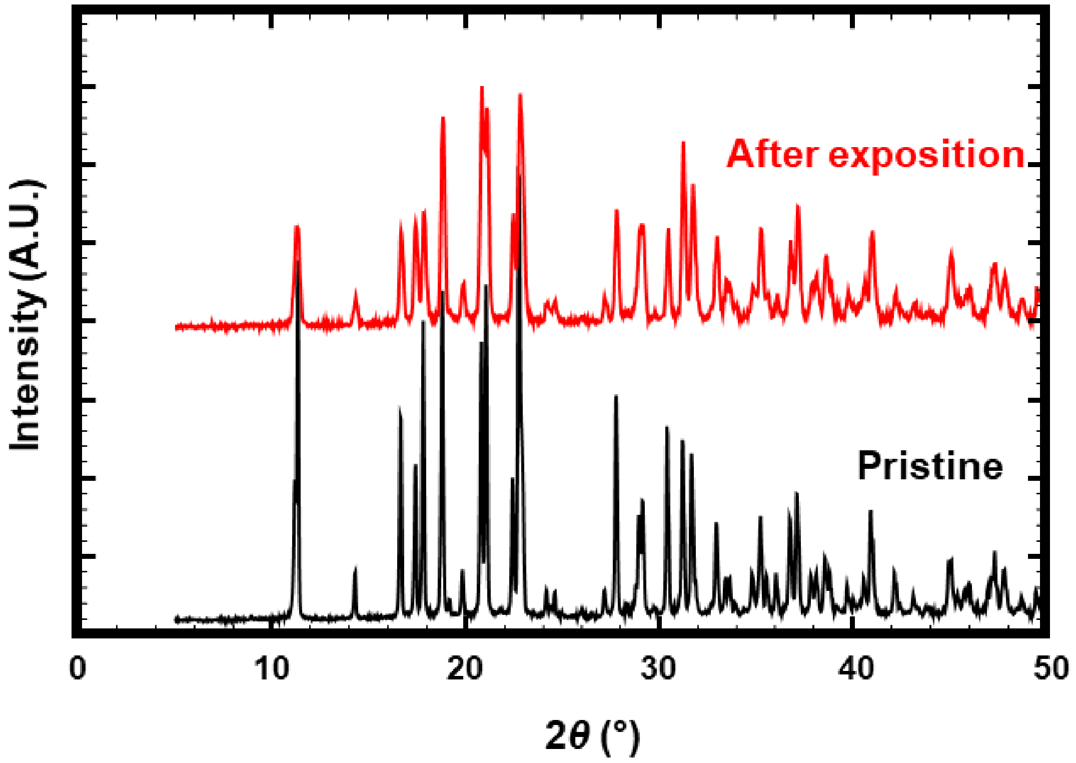

Figure S20. PXRD patterns of **GR-MOF-31** before (clack) and after (red) of the photostability studies.
